# Stem cell models of Angelman syndrome

**DOI:** 10.3389/fcell.2023.1274040

**Published:** 2023-10-19

**Authors:** João Camões dos Santos, Carolina Appleton, Francisca Cazaux Mateus, Rita Covas, Evguenia Pavlovna Bekman, Simão Teixeira da Rocha

**Affiliations:** ^1^ iBB—Institute for Bioengineering and Biosciences, Department of Bioengineering, Instituto Superior Técnico, Universidade de Lisboa, Lisbon, Portugal; ^2^ Associate Laboratory i4HB Institute for Health and Bioeconomy, Instituto Superior Técnico, Universidade de Lisboa, Lisbon, Portugal; ^3^ Department of Animal Biology, Faculdade de Ciências da Universidade de Lisboa, Lisbon, Portugal; ^4^ The Egas Moniz Center for Interdisciplinary Research (CiiEM), Caparica, Portugal

**Keywords:** Angelman syndrome (AS), genomic imprinting, UBE3A, pluripotent stem cells (PSCs), disease modeling, brain organoids, antisense oligonucleotides (ASOs)

## Abstract

Angelman syndrome (AS) is an imprinted neurodevelopmental disorder that lacks a cure, characterized by developmental delay, intellectual impairment, seizures, ataxia, and paroxysmal laughter. The condition arises due to the loss of the maternally inherited copy of the *UBE3A* gene in neurons. The paternally inherited *UBE3A* allele is unable to compensate because it is silenced by the expression of an antisense transcript (*UBE3A-ATS*) on the paternal chromosome. *UBE3A*, encoding enigmatic E3 ubiquitin ligase variants, regulates target proteins by either modifying their properties/functions or leading them to degradation through the proteasome. Over time, animal models, particularly the *Ube3a* ^mat−/pat+^ Knock-Out (KO) mice, have significantly contributed to our understanding of the molecular mechanisms underlying AS. However, a shift toward human pluripotent stem cell models (PSCs), such as human embryonic stem cells (ESCs) and induced pluripotent stem cells (iPSCs), has gained momentum. These stem cell models accurately capture human genetic and cellular characteristics, offering an alternative or a complement to animal experimentation. Human stem cells possess the remarkable ability to recapitulate neurogenesis and generate “brain-in-a-dish” models, making them valuable tools for studying neurodevelopmental disorders like AS. In this review, we provide an overview of the current state-of-the-art human stem cell models of AS and explore their potential to become the preclinical models of choice for drug screening and development, thus propelling AS therapeutic advancements and improving the lives of affected individuals.

## Introduction

Angelman Syndrome (AS) (OMIM#105830) is a rare neurodevelopmental disorder estimated to affect between 1 in 12,000 and 1 in 20,000 live births (Buiting et al., 2016). It presents a diverse symptomatology, including severe developmental delay, speech impairment, movement disorders ranging from tremors to ataxia, epilepsy, and atypical episodes of laughter and smiling. Typically, these symptoms begin to emerge between 6–9 months of age. However, it is important to note that a definitive diagnosis of AS may take some time, usually within the first 3 years of a child’s life. Several characteristic features commonly linked to AS may intersect with symptoms seen in other neurodevelopmental disorders. Therefore, a precise AS diagnosis requires validation through molecular testing (Margolis et al., 2015; Maranga et al., 2021).

AS results from the absence or deficiency of Ubiquitin Protein Ligase E3A (UBE3A) protein function in neurons. By integrating the ubiquitin-proteasome protein degradation pathway and possibly other regulatory processes, UBE3A regulates protein function and/or degradation of several specific targets through its ubiquitination activity. As a result, disruption of normal UBE3A expression is thought to affect several key neuronal processes necessary for normal synaptic function and plasticity. While *UBE3A* is biallelically expressed in most human tissues, only the maternal copy of this gene is expressed in neurons, constituting an example of a gene that is regulated by a cell type-specific form of genomic imprinting (Williams et al., 2010; Margolis et al., 2015; MedlinePlus, 2022). The lack of function of the maternal *UBE3A* copy in neurons is sufficient for the manifestation of AS.

Our current understanding of AS has been built upon studies using different models. *Postmortem* analysis of human AS tissues, animal models such as mouse, rat, or *Drosophila*, and *in vitro* cellular studies, have all furthered the knowledge of this disease and its mechanisms (Jay et al., 1991; Jiang et al., 1998; Miura et al., 2002; Wu et al., 2008; Chamberlain et al., 2010; Jiang et al., 2010; Berg et al., 2020; Dodge et al., 2020). The most useful of all has been the *Ube3a* ^mat−/pat+^ Knock-Out (KO) mouse (Jiang et al., 1998) which advanced our knowledge of the pathophysiological mechanisms of the disease. Despite overall milder symptomatology when compared to human AS individuals, this KO mouse has also been an important preclinical model for drug development. More recently, the advent of human embryonic stem cells (ESCs) and patient-derived induced Pluripotent stem cells (iPSCs) have provided the possibility of new avenues of research for AS, which overcomes some of the limitations regarding phenotype recapitulation and ethical concerns presented by animal models (reviewed in Maranga et al., 2021). Here, we aim to provide a comprehensive overview of stem cell-focused research on AS. We will delve into the advantages offered by these cellular models in terms of disease phenotyping, identification of druggable targets, and their exceptional utility as a preclinical model for drug screening.

## Research milestones in Angelman syndrome

**FIGURE 1:**
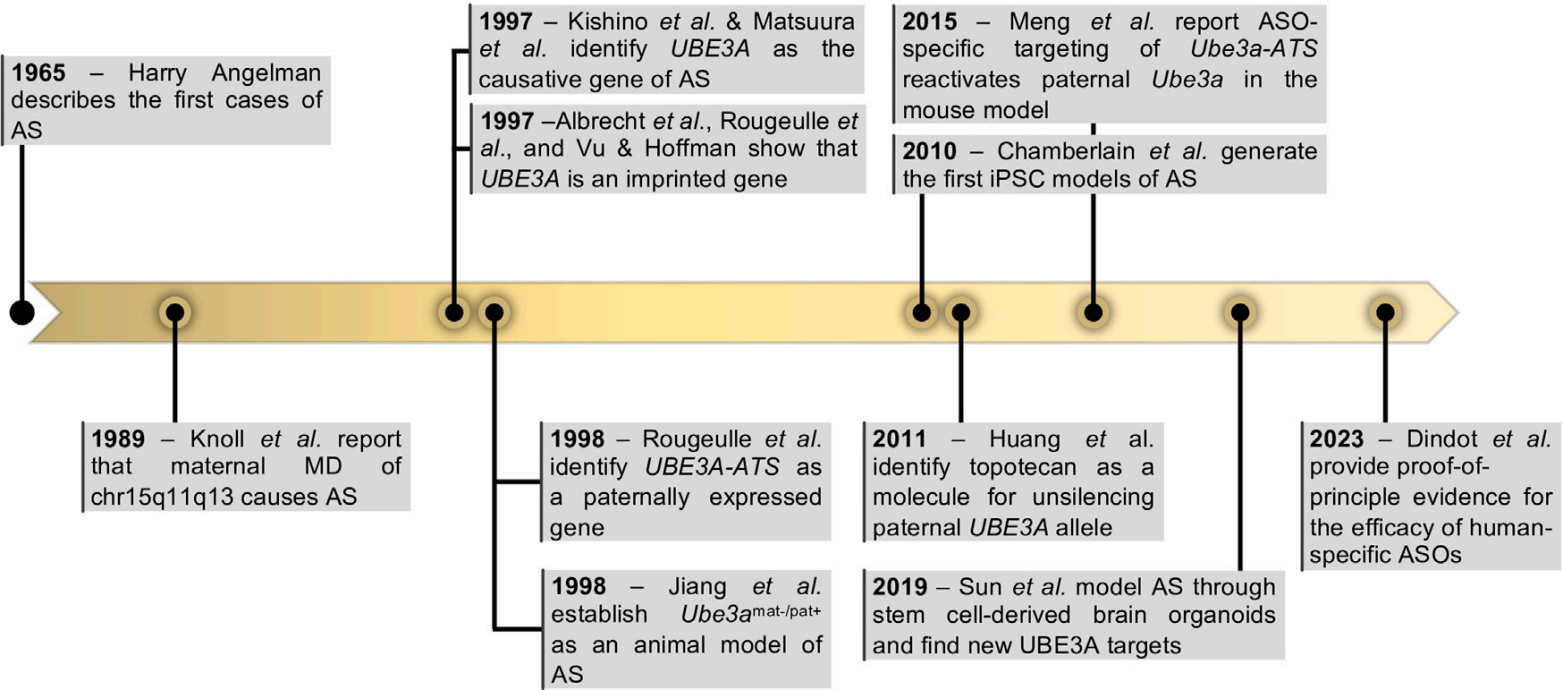
Milestones in Angelman Syndrome research. Highlighted years represent the year of the relevant publication. Abbreviations: UBE3A—Ubiquitin Protein Ligase E3A; *UBE3A-ATS*—ubiquitin-protein ligase E3A anti-sense; ASOs—antisense oligonucleotides.

AS was first described in 1965 by an English pediatrician, Harry Angelman, in *‘Puppet’ children. A report on three cases* (Figure 1). In this report, Angelman described the symptoms of three patients, which he considered similar enough to justify combining them in a *“specific group, as yet of unknown cause”*. They shared common symptoms such as depression in the occipital region of the skull, brachycephaly associated with microcephaly, severe intellectual disability, easily provoked and prolonged paroxysms of laughter, ataxia like the one observed in cerebellar deficiency and unusually protruding tongues, among other features (Angelman, 1965). Not long after, more cases had been reported and the *“Happy Puppet syndrome”* was renamed Angelman syndrome, honoring Harry Angelman as the discoverer of this new human condition (Berg and Pakula, 1972).

It was not until the late 1980s that the first genetic aberration was linked to AS. Two studies reported megabase deletions (herein named megadeletions or MDs) within the chr15q11-q13 region present in individuals with AS (Kaplan et al., 1987; Magenis et al., 1987). At the time, this was an intriguing discovery since MDs of the same region were already associated with Prader-Willi syndrome (PWS), a very different condition characterized by mild-to-moderate intellectual impairment, constant feeling of hunger, and obesity (OMIM#176270). The mystery was later solved by the discovery that the parental origin of the chr15q11-q13 MD dictated the disease presentation: inheritance of the paternal deletion results in PWS, while maternal deletion results in AS (Knoll et al., 1989) (Figure 1). These findings suggested that chr15q11-q13 was regulated by genomic imprinting, an epigenetic phenomenon that regulates monoallelic expression of genes according to their parental origin. Therefore, PWS and AS were proposed to be the first examples of imprinting disorders (Hall, 1990; Williams et al., 1990). This was further supported by the discovery that inheritance of two paternal chromosomes 15 (patUPD15) also causes AS (Malcolm et al., 1991), while maternal uniparental disomy was a frequent cause of PWS (Butler, 1983). Once the importance of DNA methylation in regulating genomic imprinting was established (Li et al., 1993), several reports soon found differential methylated regions (DMRs) between the two parental alleles at the chr15q11-q13 region (Dittrich et al., 1992; Driscoll et al., 1992; Clayton-Smith et al., 1993). Unusual and contrasting DNA methylation patterns were then reported in both PWS and AS patients with no obvious genetic abnormality (Reis et al., 1994). This revealed that the abnormal establishment of DNA methylation at DMRs was sufficient to cause these imprinting diseases. The important genetic elements, also known as imprinting centers (IC), were later mapped to a region including *“D15S63 (PW71) and SNRPN”* thanks to microdeletions found in AS and PWS individuals and further fine-tuned to two regions now known as AS and PWS ICs (AS-IC and PWS-IC). PWS-IC, also known as *SNURF* TSS-DMR, was then confirmed to hold a DMR inheriting the methylation mark only from the maternal germline (Buiting et al., 1995; Shemer et al., 2000).

**FIGURE 2:**
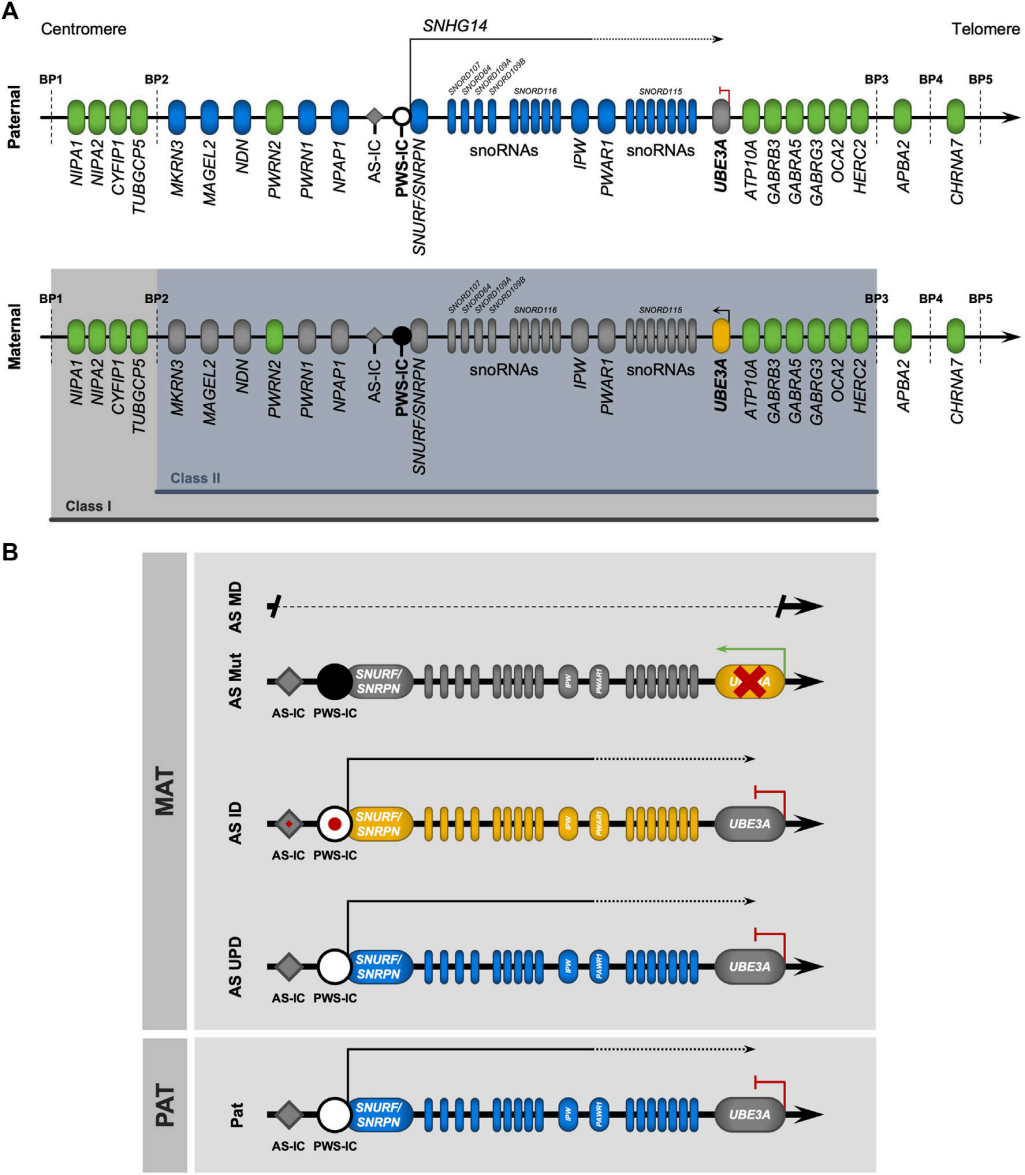
The (epi)genetics of Angelman syndrome. **(A)** Genomic map of the chr15q11-q13 region. Parental-of-origin specific DNA methylation (black circle) occurs on the CpG-rich locus known as the Prader-Willi imprinting center (PWS-IC), only on the maternal allele. From the unmethylated paternal allele, PWS-IC serves as a promotor for a large transcription unit (small nucleolar host gene 14, *SNHG14*) containing the transcripts of some genes (*SNURF/SNURPN*, *IPW*) and a group of C/D small nucleolar RNAs (snoRNAs) with the *SNORD116* host transcript, expressed only from the paternal allele (in blue). In neurons, the loss of an insulator element at the *IPW* and *PWAR1* locus results in the extension of *SNHG14*, which now contains *SNORD115* (also containing C/D snoRNAs) and *UBE3A-ATS*, an antisense transcript that silences the paternal *UBE3A* allele by transcriptional interference. Thus, *UBE3A* is only expressed from the maternal allele (in yellow) in neurons. Biallelically expressed genes are represented in green. The two most common types of large deletions that lead to Angelman syndrome (AS) are highlighted between breakpoints (BP) BP1-BP3 (class I) and BP2-BP3 (class II). **(B)** Genomic map of the (epi)genetic causes of Angelman syndrome. AS is caused by four main causes relating to the maternal chr15, all leading to a loss-of-function of UBE3A: megadeletions in the chr15q11-q13 region (AS MD); deleterious mutations in the *UBE3A* allele (AS Mut); imprinting defects (red spot) at the PWS-IC locus causing an absence of DNA methylation (AS ID); and paternal uniparental disomy of chr15 (AS UPD).

While it was becoming evident that AS was an imprinting disorder affecting the chr15q11-13 region, the causing gene(s) were yet to be identified. In 1997, two back-to-back publications undoubtedly pinpointed *UBE3A* as the gene implicated in AS (Kishino et al., 1997; Matsuura et al., 1997) (Figure 1). Both studies report distinct mutations in the *UBE3A* gene as the cause of AS in non-MD/non-UPD/non-imprinting defect AS individuals. Shortly after, *UBE3A* was confirmed to be an imprinted gene, expressed only from the maternal allele (Figure 1). However, in contrast to most genes known at the time, imprinting of *UBE3A* was restricted to the brain (Albrecht et al., 1997; Rougeulle et al., 1997; Vu and Hoffman, 1997). In 1998, Rougeulle *et al.* identified the antisense non-coding transcript of human *UBE3A*, commonly referred today as *UBE3A-ATS*, which was reciprocally imprinted, being expressed only from the paternal allele in the brain (Rougeulle et al., 1998) (Figure 1). This antisense RNA was later shown to belong to a large polycistronic transcript originated from the unmethylated paternally inherited PWS-IC region and encoding several distinct transcripts including *SNRPN/SNURF*, *IPW*, *PWAR1* and tandemly repeated C/D *snoRNA* genes, besides the antisense RNA to *UBE3A* (Runte et al., 2001) (Figure 2A). Since its discovery, *UBE3-ATS* has been anticipated to be a putative regulator of paternal *UBE3A* silencing (Chamberlain and Brannan, 2001). Formal proof of that was first shown thanks to the addition of a transcription termination cassette that halted *UBE3A-ATS* expression and resulted in the unsilencing of *UBE3A* from the paternal allele (Meng et al., 2012).

Advances in AS research would have only been possible with the use of a variety of research models. Through the years, researchers have privileged the use of mouse models with AS-like phenotypes. The first AS mouse model was described by Cattanach *et al.* in 1997 and consisted of a paternal duplication of the murine homologous region of the human chr15q11-q13 (Cattanach et al., 1997). Although these mice exhibited AS traits, they needed complex breeding schemes and were never extensively used for dissecting pathophysiological mechanisms of disease. With the discovery that loss of function of the maternal copy of *UBE3A* gene causes AS, Jiang *et al.* created the *Ube3a* ^mat−/pat+^ KO mice, with a deletion on the maternally inherited *Ube3a* allele, which presents ataxia, inducible seizures, and sleep alterations, all features shown by AS individuals (Jiang et al., 1998; Colas et al., 2005; Dindot et al., 2007). Other AS mouse models have been generated with similar phenotypes (Miura et al., 2002; Jiang et al., 2010), but the original *Ube3a* ^mat−/pat+^ mice from Jiang et al. (1998) has remained the preferential AS mouse model used by researchers. Other important mouse models comprise the *Ube3a* ^Stop/p+^; *Cre* ^*ERT+*^ mouse allowing for temporal control of *Ube3a* reinstatement to discern the critical developmental time windows for therapeutic intervention (Silva-Santos et al., 2015; Gu et al., 2018; Rotaru et al., 2023), the conditional *Ube3a* floxed allele, *Ube3a* ^fl/+^ (Bruinsma et al., 2015; Gu et al., 2018) used to investigate the cell/region-specific contribution to the disease phenotype or the *Ube3a* ^m+/p*YFP*^ knock-in mice (Dindot et al., 2007) which provide a useful read-out for drug screening strategies aiming at unsilencing paternal *Ube3a* allele as a therapeutic option to treat AS (Huang et al., 2012; Meng et al., 2015). Recently, AS rat models with a complete KO of the maternal *Ube3a* copy have been developed (Berg et al., 2020; Dodge et al., 2020; Born et al., 2021). These larger rodents, while sharing phenotypic similarities with *Ube3a* ^mat−/pat+^ KO mice, displayed distinctive behaviors and previously unseen changes in neuroanatomy (Berg et al., 2020; Dodge et al., 2020; Born et al., 2021), bringing an added value to AS research.

After the seminal studies from Thomson et al. (1998) on the derivation of human embryonic stem cells (ESCs) and from Yamanaka and others in the mid-2000s (Takahashi and Yamanaka, 2006; Takahashi et al., 2007) on the generation of induced pluripotent stem cells (iPSCs), PSC models emerged as alternative humanized and personalized cellular systems for disease modeling. The first AS iPSC models were soon generated (Chamberlain et al., 2010), initiating a new era of research on AS based on the use of stem cell-derived neurons and, later on, brain organoid models to reveal new pathophysiologic mechanisms of the disease, find new ubiquitination targets of UBE3A and validate potential therapeutic approaches (Fink et al., 2017; Sun et al., 2019; Pandya et al., 2021; Dindot et al., 2023).

Disease management for AS relies on approaches that ameliorate the most detrimental symptoms such as seizures or sleep abnormalities. None of these therapeutic interventions targets the cause of the disease, which is the loss of function of the *UBE3A* gene. In recent years, many hopes have been put on emerging strategies to reinstate *UBE3A* expression as a therapeutic option. The favored strategy has not been the ectopic expression of *UBE3A* (Daily et al., 2011), amid fears of elevating the dosage of *UBE3A*, a known cause of the autism and epilepsy-related Dup15q syndrome (Lusk et al., 2021), but rather the reactivation of the intact, albeit silenced paternal copy of the *UBE3A* gene. Using primary cortical neurons from *Ube3a* ^m+/p*YFP*^ mice, a study identified inhibitors of topoisomerase I and II as molecules that reactivate the paternal *Ube3a*-YFP allele (Huang et al., 2012). The most promising compound was topotecan, a clinical-grade topoisomerase I inhibitor, that was shown to reactivate paternal *Ube3a* through the reduction of transcription of the polycistronic transcription unit containing *Ube3a-ATS*. Although topotecan has a generalized effect on long genes associated with autism (King et al., 2013) that may halt its widespread use as a therapeutic agent to treat AS, this seminal study showed that pharmacological perturbation of *UBE3A-ATS* transcription is a feasible approach to reinstate *UBE3A* expression in AS individuals. This idea was further explored by Meng et al. (2015) who screened for antisense oligonucleotides (ASOs) capable of specifically downregulating *Ube3a-ATS.* This downregulation was able to activate the paternal *Ube3a* allele (Meng et al., 2015) resulting in phenotypic rescue of cognitive and behavior deficits in the *Ube3a* ^mat−/pat+^ KO mouse (Meng et al., 2015; Milazzo et al., 2021). This study was later translated to human cells with the identification of primate-specific ASOs able to reactive paternal *UBE3A* not only in iPSC-derived neurons *in vitro* but also *in vivo* by lumbar puncture in cynomolgus macaques (Dindot et al., 2023). These findings support the molecular basis for an ongoing clinical trial (ClinicalTrials.gov, NCT04259281), soon followed by another one also using oligonucleotide-based therapeutics (NCT04428281). Other attempts to perturb UB*E3A-ATS* transcript, by taking advantage of the CRISPR/Cas9 genetic editing technology, are under development with promising results achieved in the mouse model (Wolter et al., 2020; Schmid et al., 2021; Li et al., 2023).

More than 50 years after the initial description of the *‘Puppet’ children* by Dr. H. Angelman, a promising therapy targeting the molecular cause of the disease has reached the clinical stage (Dindot et al., 2023). However, many challenges in AS research and treatment remain, justifying continuous efforts in investigating further the multiple aspects of this disease for which stem cell models are becoming increasingly important as research tools.

## Clinical hallmarks of Angelman syndrome

From the 1960s through the 1990s several studies further characterized the symptoms of the disorder first reported by Dr. Harry Angelman (Bower and Jeavons, 1967; Berg and Pakula, 1972; Williams et al., 1982; Robb et al., 1989; Dickinson et al., 1990). This ultimately led to a clinical consensus concerning the symptomatology of AS individuals. The diverse symptoms observed in AS individuals have been divided into three categories: consistent, frequent, and associated features. Consistent features are those present in all AS patients and include functionally severe developmental delay, a movement or balance disorder (usually ataxia), a combination of frequent laughter and smiling and hypermotoric behavior, and absent or impaired speech. Frequent characteristics are present in ≥80% of AS patients, and include microcephaly, early onset seizures, and a specific and abnormal electroencephalogram pattern. The remaining shared traits, affecting from 20% to 80% of patients, include the occipital groove and protruding tongue observed by Harry Angelman, as well as a wide variety of symptoms, among them feeding problems, prognathia, an uplifted and flexed arm position during ambulation, wide-based gait, abnormal sleep cycles and food-related behaviors, and attraction/fascination with water (Williams et al., 2006). Some of these symptoms become apparent as early as 6–9 months old, with most AS diagnoses happening between 9 months and 6 years of age (Mayo Clinic, 2022; NHS, 2023). AS patients have a reasonably long lifespan, with some patients living past 70 years of age. Reduced lifespan of some patients is mostly associated with epilepsy (severe convulsions) and lack of balance/coordination (ambulatory accidents), combined with a hyperactive and exploratory personality often seen in children with AS (Buiting et al., 2016).

AS shares similarities with other neurodevelopmental disorders with mutations in other genes, which could cause difficulty in early diagnosis. These include Rett syndrome (*MECP2*, OMIM#312750), early infantile epileptic encephalopathy (*CDKL5*, OMIM#300672), *FOXG1* syndrome (OMIM#613454), Christianson syndrome (*SLC9A6*, OMIM#300243), or Pitt-Hopkins syndrome (*TCF4*, OMIM#610954) (McKnight et al., 2022). Given the overlapping manifestations, a definitive diagnosis relies on molecular testing, which in the case of AS may need several independent tests depending on the molecular cause of the disease.

## Molecular causes of Angelman syndrome

The chr15q11-q13 region, where the *UBE3A* gene is located, is regulated by genomic imprinting (Figure 2A). This epigenetic mechanism of gene regulation selectively silences one of the two parental alleles, resulting in a parental-of-origin monoallelic expression of the imprinted genes (reviewed in da Rocha and Gendrel, 2019). Imprinting regulation in the chr15q11-q13 region is ensured by the PWS-IC, which is characterized by a dense CpG sequence with maternal allele-specific DNA methylation, established in the germline (da Rocha and Gendrel, 2019). Methylation of the PWS-IC in the maternal germline is established by the transcription of upstream exons of the *SNURF/SNRPN* bicistronic gene, driven by a promoter element known as AS-IC, which induces transcription-associated CpG methylation at the maternal PWS-IC (Horsthemke and Wagstaff, 2008). The unmethylated paternal PWS-IC serves as a promoter of a large polycistronic transcription unit, also known as *SNHG14* (small nucleolar RNA host gene 14), exclusively expressed from the paternal allele. *SNHG14* encodes the bicistronic *SNURF/SNRPN* gene pair and several long and small RNAs. These include *IPW*, *PWAR1* long noncoding RNAs (lncRNAs), and tandem-repeated C/D *snoRNA* genes clustered in two domains, known as *SNORD116*, which is ubiquitously expressed, and *SNORD115*. At the 3’ end tip of the *SNHG14* transcript unit sits the *UBE3A-ATS* lncRNA which overlaps with the *UBE3A* gene (reviewed in Maranga et al., 2020) (Figure 2A). In non-neuronal cells, *UBE3A* is biallelically expressed, as *UBE3A-ATS* is absent. However, in neurons, loss of an insulator element at the *IPW* and *PWAR1* genes, composed of poly(A), conserved sites, and CTCF (CCCTC-binding factor) binding motifs, results in the extension of the *SNHG14* transcript (Vu and Hoffman, 1997; Hsiao et al., 2019), that, in its full form, includes *SNORD115* and *UBE3A-ATS*, which silences the paternal allele of *UBE3A* by transcription interference (Meng et al., 2012; Meng et al., 2013). As such, *UBE3A* expression in neurons is exclusively ensured by the maternal allele, which leads to AS when absent, not expressed, or mutated.

Loss-of-function of maternal *UBE3A* may result from four main (epi)genetic defects (Figure 2B), with varying degrees of disease manifestation and severity (reviewed in Maranga et al., 2020; Yang et al., 2021): megadeletions in the maternal chr15q11-q13 region—AS MD (60%–70%); deleterious mutations in the maternal *UBE3A* gene—AS Mut (10%); paternal uniparental disomy of chromosome 15—AS UPD (10%); imprinting defects on the maternal PWS-IC—AS ID (3%–5%). Some cases (<10%), despite having AS-like clinical diagnosis, are not attributed to any of the four known (epi)genetic causes and may arise from genetic abnormalities in other genes (Aguilera et al., 2021) or are misdiagnosed by another neurodevelopmental disorder with similar disease presentation (McKnight et al., 2022).

Significant heterogeneity in disease severity is observed across the different (epi)genetic origins of AS (Keute et al., 2021). AS MD presents the most severe manifestations, with stark development delays, as well as more frequent and grave seizures, when compared to the other causes (Gentile et al., 2010). Hypopigmentation is also a characteristic of individuals carrying MDs (Luk and Lo, 2016), likely associated with the haploinsufficiency of the *OCA2* and *GABRB3* genes (Delahanty et al., 2016) (Figure 2A). The vast majority of AS MD cases (95%) (Yang et al., 2021) falls under either class I (BP1-BP3, ∼6 Mb/16 genes) or class II (BP2-BP3, ∼5 Mb/12 genes) MDs (Figures 2A, B). In these cases, the absent chromosomal region includes *UBE3A* and several other imprinted and non-imprinted genes. While paternally imprinted genes remain unaffected, non-imprinted genes have half of their normal expression levels. Besides *UBE3A*, class I and II megadeletions cause the absence of the maternally inherited copies of three GABA_A_ receptor subunit genes (*GABRB3*, *GABRA5,* and *GABRG3*), which are genes implicated in neuronal development, synaptic function, and epilepsy (Tang et al., 2021). Another gene absent in both megadeletions is *HERC2.* This gene encodes for a protein that interacts with UBE3A and is also involved in ubiquitination (Galligan et al., 2015). Defects on this gene cause an autosomal recessive AS-like syndrome named intellectual developmental disorder, autosomal recessive 38 (OMIM#615516) (Kühnle et al., 2011; Puffenberger et al., 2012; Cubillos-Rojas et al., 2016). Class I deletions have an additional deleted region (BP1-BP2) that encompasses four evolutionarily conserved genes (*NIPA1*, *NIPA2*, *CYF1P1*, and *TUBGCP5*) involved in brain development and function (Burnside et al., 2011; Vanlerberghe et al., 2015). The difference in deletion size suggests class I MD should result in the most severe phenotypes, with some evidence in favor (Valente et al., 2013). However, analyses show disagreement, with recent data suggesting only minor phenotypic differences between individuals carrying class I or II MDs, as measured by scales of development, in terms of cognitive ability, motor, social, and communication skills (Keute et al., 2021). Further comparative studies may be needed for a definitive answer on the potential differences between class I and II MDs.

Up to ten percent of AS cases are AS UPD that lack the maternal copy of chr15, having instead two paternal copies of this chromosome. This results in the complete silencing of *UBE3A*, and results in overexpression of paternally expressed imprinted genes, such as *SNURF/SNRPN*, *IPW*, *SNORD115/116*, and *UBE3A-ATS* (Figure 2B). These patients have milder disease manifestations compared to AS MD cases, with less prevalence and severity of seizures, but still have severe development delay and more pronounced sleep problems (Gentile et al., 2010; Yang et al., 2021). Interestingly, AS UPD individuals tend to present hyperphagia and a higher risk of obesity (Brennan et al., 2015).

In individuals with AS ID, the maternal copy of the chr15q11-q13 region suffers an epigenotype switch to become indistinguishable from the paternal copy (Figure 2B). In other words, the PWS-IC of both chromosomes lacks DNA methylation, thus resulting in expression from the PWS-IC region and silencing of not only the paternal but also the maternal copy of *UBE3A*. The ID arises from a failure to establish the maternal imprint at PWS-IC during female germline development, but the reasons for this failure are not always clear. In certain instances, imprinting defects on the PWS-IC arise from mutations and microdeletions at the AS-IC or PWS-IC affecting the ability to create the imprint in the maternal germline (Horsthemke and Buiting, 2006; Beygo et al., 2020). At the molecular level, AS ID and AS UPD share the same transcriptional profile at chr15q11-q13 (Figure 2B), with the most relevant difference being the fact that AS UPD are homozygous for all loci on chr15 when this is not the case for AS ID. At the clinical level, AS ID individuals share similar phenotypes and disease progression with individuals with AS UPD, often showing milder characteristic impairments (Gentile et al., 2010; Yang et al., 2021). Additionally, similarly to AS UPD individuals, AS ID individuals also tend to have hyperphagia and an increased risk of obesity (Brennan et al., 2015).

There is a wide range of reported adverse mutations in the maternal copy of the *UBE3A* gene that cause AS (AS Mut), most of which are nonsense mutations that lead to frameshifts and premature stop codons (Sadikovic et al., 2014). Besides mutations that result in the truncation of the maternal *UBE3A* transcript, loss of function of UBE3A may also result from missense mutations affecting active domains and protein stability (Beasley et al., 2020), intracellular localization (Bossuyt et al., 2021), and even gain of function (Weston et al., 2021), although the latter is often associated with Dup15q syndrome and not AS (Copping et al., 2017; Xing et al., 2023). AS individuals carrying *UBE3A* mutations often present the mildest phenotypes out of the four (epi)genetic causes, with less pronounced development delay (Gentile et al., 2010; Yang et al., 2021), as seen by clinical scales of development in infancy (Keute et al., 2021). Epileptic episodes are an exception, as these tend to be more severe for AS Mut than AS UPD or AS ID individuals (Luk and Lo, 2016; Yang et al., 2021).

Molecular diagnosis of AS is important not only to rule out other clinically similar diseases but also to understand the (epi)genetic cause underlying the clinical AS diagnosis, which could impact disease management. Given the distinct (epi)genetic causes of AS, more than one molecular diagnostic test is needed (Bird, 2014; Maranga et al., 2020; Yang et al., 2021). If an individual has clinical symptoms of AS, the first step is to evaluate the DNA methylation status at the PWS-IC locus, usually by methylation-specific PCR or methylation-specific multiplex ligation-dependent probe (MS-MPLA), the latter of which also detects large deletions in genomic DNA. If normal DNA methylation is detected, the *UBE3A* gene is sequenced to screen for potential pathogenic mutations (AS Mut), and if negative, AS-like syndromes should be investigated. Instead, if DNA methylation is anomalous, the second step is to determine whether or not the patient has a large deletion of the chr15q11-13 region, by MPLA, fluorescence *in situ* hybridization (FISH) for chr15q11-q13, or a comparative genomic hybridization array (array CGH). If a maternal MD is detected, the patient has AS MD. If instead, no megadeletion is present, microsatellites or nucleotide polymorphism markers for chr15 are used to assess if the patient has AS UPD (only paternal markers) or AS ID (maternal and paternal markers).

## Pathophysiological mechanisms of Angelman syndrome

**FIGURE 3:**
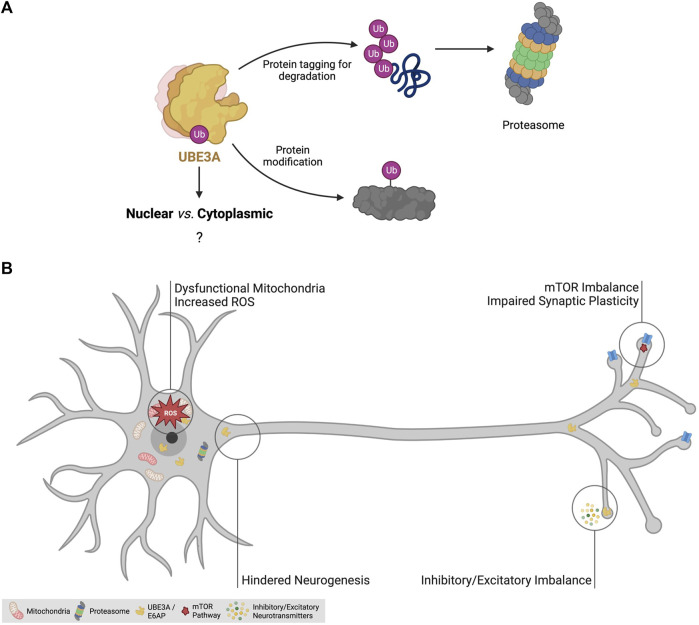
The disease mechanisms of Angelman syndrome. **(A)** Roles of ubiquitin-protein ligase E3A (UBE3A). UBE3A is an E3 ligase responsible for the addition of ubiquitin to specific targets. Polyubiquitination is known to be a mechanism to tag proteins for proteasomal degradation. UBE3A is also known to mediate monoubiquitination to modulate the protein activity of certain targets. The function of its three isoforms and the role of nuclear and cytoplasmic UBE3A remain elusive. **(B)** Identified dysregulated cellular processes in AS models. AS mouse models have been crucial to unravel pathophysiological mechanisms of the disease and have allowed the identification of signaling pathway imbalance, dysfunctional mitochondria, increased oxidative stress, impairments in neurogenesis and synaptic plasticity, and inhibitory/excitatory imbalance as features of the AS brain, likely contributing to the clinical manifestations of the disease. Abbreviations: Ub - ubiquitination; ROS—reactive oxygen species; mTOR—mechanistic target of rapamycin pathway. Created with Biorender.com.

UBE3A, also known as E6-associated protein (E6AP), is a 100 kDa protein that tags proteins for proteasomal degradation or for acquiring novel properties through target-specific ubiquitination (Huibregtse et al., 1993; Avagliano Trezza et al., 2021) (Figure 3A). Ubiquitination is a type of post-translational modification mediated by a three-step enzyme cascade, E1-E2-E3 [reviewed in Damgaard (2021)], that adds the small peptide ubiquitin (Ub) to a target protein. The first enzyme (E1) is responsible for the activation of free ubiquitin, in an ATP-dependent manner, and then transfer it to the E2 enzyme. Finally, the E2-Ub complex mediates the transfer of Ub to the E3 enzymes, which then covalently links it to specific target proteins, mediated by the HECT (Homologous to the E6-AP Carboxyl Terminus) domain of E3. As the final mediators of the cascade, E3 ligases are the most specific of the three types of enzymes, having individualized targets, and are abundant in the human genome (Medvar et al., 2016), with UBE3A being one example. Although first identified as a marker for proteasomal degradation, ubiquitination is a cellular tool for the regulation of protein activity and is involved in many cellular processes including cell cycle control, apoptosis, signal transduction, intracellular traffic, DNA repair, and more (Damgaard, 2021; Mathieu et al., 2021).

Given the pivotal role of UBE3A loss-of-function in AS, its downstream targets may be relevant to uncover pathophysiological mechanisms and potential therapeutic targets for the disease. Examples of these ubiquitination targets include tumor suppressor p53 (Huibregtse et al., 1993), synaptic regulator ARC (Greer et al., 2010), small-conductance potassium channel 2 (SK2) (Sun and Zhu, 2015), voltage-dependent big potassium (BK) (Sun et al., 2019), and RPH3A (Avagliano Trezza et al., 2021). However, some targets may not actually be direct targets of UBE3A. Such is the example of synaptic regulator ARC, which was initially reported as a ubiquitination target of UBE3A (Greer et al., 2010), but more recent data suggest it is not a UBE3A target (Pastuzyn and Shepherd, 2017). As such, rigorous and controlled ubiquitination experiments should be performed to confirm a potential candidate as a ubiquitination target of UBE3A.

UBE3A has three known isoforms, with isoform 1 being the most abundant, and all three can be localized in both the cytoplasm and the nucleus in human ESCs and ESC-derived neurons (Sirois et al., 2020). This contrasts with the situation in the mouse where isoforms with clear nuclear and cytoplasmic localization have been found (Avagliano Trezza et al., 2019). Interestingly, KO mice for the nuclear isoform of *Ube3a* showed severe behavior impairments and synaptic defects, while KO mice for the cytoplasmic isoform were asymptomatic (Avagliano Trezza et al., 2019). As such, these results suggest loss of nuclear activity of UBE3A may be critical in AS (Sirois et al., 2020; Bossuyt et al., 2021), but its function remains elusive.

Over the decades, many studies have identified disrupted molecular pathways and mechanisms in the hippocampus, cortex, striatum (Rotaru et al., 2023), and cerebellum of AS models [reviewed in Maranga et al. (2020)] (Figure 3B). The dysregulation of the mechanistic target of rapamycin (mTOR) pathway in AS mice has been widely reported (Sun and Liu, 2015; Sun et al., 2016; Pastuzyn and Shepherd, 2017). The mTOR pathway, comprising the two complex families mTORC1 and mTORC2, is implicated in a plethora of cellular processes such as cell growth, lipid synthesis, mitochondria biogenesis, and apoptosis. This pathway is important for neuronal activity as it regulates autophagy, lysosome biogenesis, and actin dynamics (Costa-Mattioli and Monteggia, 2013; Liu and Sabatini, 2020). In the brain of AS mice, an imbalance of the mTOR pathway, expressed in the form of increased mTORC1 activity and decreased mTORC2 activity, leads to increased levels of the ARC protein and impaired actin remodeling (Sun and Liu, 2015; Sun et al., 2016; Pastuzyn and Shepherd, 2017), potentially contributing to the cognitive and behavioral impairments observed in AS.

Mitochondrial dysfunction and increased oxidative stress have been described as hallmarks of AS animal models (Su et al., 2011; Llewellyn et al., 2015; Santini et al., 2015; Berkowitz et al., 2017). In fact, impairments in oxidative phosphorylation and increased levels of reactive oxygen species (ROS) are associated with several neurodevelopmental and neurodegenerative disorders, appearing as a common theme in brain diseases (Keating, 2008; Norat et al., 2020; Anitha et al., 2023). In AS models, increased levels of ROS have been linked to compromised hippocampal synaptic function (Su et al., 2011; Santini et al., 2015). They affect neurodevelopment by causing mitochondrial malfunctioning in neural precursor cells leading to excessive ROS and increased apoptosis (Simchi et al., 2023). However, how UBE3A is influencing mitochondrial redox homeostasis is still unclear and might prove insightful to better understand the pathophysiology of AS and other neurodevelopmental diseases.

Epilepsy is another consistent feature of AS and it is hypothesized to result from dysfunctional GABAergic circuitry (Gu et al., 2018), likely suggesting a distinctive imbalance between inhibitory and excitatory signals (Yashiro et al., 2009; Wallace et al., 2012; Fink et al., 2017; Rotaru et al., 2018). Deficits in inhibitory and excitatory neuronal circuits have been reported in the AS mouse brain (Egawa et al., 2012; Wallace et al., 2012; Rotaru et al., 2018), suggested to be a consequence of impaired synaptic plasticity and dendritic spine formation. Coupled with this, long-term potentiation (LTP), an example of synaptic plasticity associated with learning and memory, has been shown to be impaired in AS mice (Kaphzan et al., 2011; Sun and Liu, 2015). In short, over the years, many studies have pointed out several impaired cellular mechanisms and neuronal functions, including synaptic plasticity, mitochondrial dysfunction, increased oxidative stress, and excitatory/inhibitory imbalance, that overall contribute to the pathophysiology of AS (Maranga et al., 2020) (Figure 3B).

## Pluripotent stem cell models of Angelman syndrome

Animal models of AS have been pivotal for the understanding of disease mechanisms and as preclinical models to advance new therapeutics [reviewed in Maranga et al. (2020)]. However, they have several limitations when used to model human diseases (Kelley and Pașca, 2022). First, they exhibit significant biological differences with humans and may not reflect accurately the disease phenotypes and/or differ in their responses to therapeutic agents. Second, mouse models do not capture the genetic and phenotypic heterogeneity seen in the human population. This is well illustrated in the case of AS where different molecular causes give rise to symptoms of diverse severity. The most commonly used AS animal models represent the loss of function of *Ube3a* alone (Jiang et al., 1998; Miura et al., 2002; Berg et al., 2020; Dodge et al., 2020). However, the majority of AS individuals have MDs associated with the loss of *UBE3A* plus haploinsufficiency of dozens of genes. This, in part, could also explain the milder phenotypes observed in these animal models when compared to AS individuals. Third, the use of animals for experimentation raises ethical concerns due to the potential harm inflicted on them. For these reasons, alternative research models that can complement or replace the use of animals are very welcomed. Human ESCs and iPSCs represent potent tools for bridging gaps in existing animal-based disease models, yielding supplementary insights into human biology by exploring human neurodevelopment *in vitro*. Nevertheless, stem cell models also raise ethical considerations, notably stemming from the embryonic provenance of hESCs and the potential for misuse of human PSCs, and they do not supplant the indispensable role of animal models in behavior assessment.

In the last decade, a great effort has been made to generate PSC models of AS. Apart from the derivation of iPSCs from AS patients, advances are being made in the genetic editing of *UBE3A* in iPSCs or ESCs (Tables 1, 2). Stem cell models have also been engineered to study the molecular mechanisms regulating imprinting at the PWS/AS cluster (Hsiao et al., 2019). The first stem models of AS were iPSCs derived from skin fibroblasts of male and female individuals with AS MD (Chamberlain et al., 2010). In the past decade, many other AS iPSC lines were derived from different somatic origins (fibroblasts, peripheral blood mononuclear cells, or B lymphocytes) and encompassing the four main molecular causes of the disease (Table 1). Initially, these iPSCs were generated using retroviral/lentiviral vectors that integrated the Yamanaka factors or analogs into the genome (Chamberlain et al., 2010; Stanurova et al., 2016; Fink et al., 2017; Pólvora-Brandão et al., 2018). More recently, iPSC models have been generated using non-integrative methods such as Sendai viruses or episomal vectors (Takahashi et al., 2017; Neureiter et al., 2018; Niki et al., 2019; Li et al., 2022; Maranga et al., 2022).

**TABLE 1:** Angelman syndrome individual-derived induced pluripotent stem cell lines (iPSCs). The table features the reference article where each iPSC line was generated, the biological sex of the individual with Angelman syndrome (AS) from which the line was derived, cell donor source used for reprogramming, details on the molecular causes of AS and the method used for reprogramming. n.s. Means not specified; * information of the exact mutation could not be retrieved; ** information on whether the megadeletion (MD) was class I or II or other could not be found; Abbreviations: MD - megadeletion; *OSKM* - short for the Yamanaka cocktail composed of *OCT4*, *SOX2*, *KLF4,* and *c-MYC*); Mut - mutation; UPD - uniparental disomy; ID - imprinting defect.

References	Biological sex	Cell donor source	The molecular cause of AS	Method of reprogramming
Chamberlain et al. (2010)	Male and Female	Fibroblasts	Class II MD of the maternal chr15q11-q13	Retroviral vectors for *OSKM* and *LIN28*
Stanurova et al. (2016)	Female	Fibroblasts	*UBE3A* Mut: in-frame 3bp deletion (p.G538del, c.1613-1615delGAG)	Lentiviral excisable vector for *OSKM*
Fink et al. (2017)	Male	n.s.	*UBE3A* frameshift mutation (2 bp deletion)*	Retroviral and lentiviral vectors for *OSKM* and *LIN28*
Takahashi et al. (2017)	Male	B lymphocytes	Paternal UPD for chr15	Episomal vectors for *OSKM*
Pólvora-Brandão et al. (2018)	Female	Fibroblasts	Class II MD of the maternal chr15q11-q13	Lentiviral vector for *OSKM*
Neureiter et al. (2018)	Female	Fibroblasts	ID at the PWS-IC	Episomal vectors for *OSKM, LIN28,* and a shRNA against p53
Niki et al. (2019)	Female	PBMCs	MD of chr15q11.2–q13**	Episomal vectors for *OSKM, LIN28, EBNA1* and p53 carboxy-terminal dominant-negative fragment
Maranga et al. (2022)	Female	Fibroblasts	Class II MD of the maternal chr15q11-q13	Sendai virus for *OSKM*
Li et al. (2022)	Female	PBMCs	*UBE3A* Mut: missense mutation (p.Asp563GLy, c.1688 A > G)	Episomal vectors for pCE-hOCT3/4, pCE-hSK, pCE-hUL, pCE-mP53DD and pCXB-EBNA1

**TABLE 2:** *UBE3A* Knock-Out (KO) stem cell models. The table displays the information on the reference article, cell line, gene editing approach, and genetic modification for each *UBE3A* Knock-Out (KO) stem cell model. Abbreviations: iPSC - induced pluripotent stem cell; CRISPR/Cas9 - clustered regularly interspaced short palindromic repeats/CRISPR-associated protein 9; KO - Knock-Out; TSS - transcription start site; ESC - embryonic stem cell; ssODN - single-stranded oligodeoxynucleotides; Iso1 - isoform 1; Iso2 - isoform 2; Iso3 -isoform 3. * The reference of the original non-AS iPSC could not be retrieved.

References	Cell line	Gene editing approach	Genetic modification
Fink et al. (2017)	iPSC*	CRISPR/Cas9	*UBE3A* KO (1 bp G) insertion at the TSS)
Sun et al. (2019)	H1 and H9 ESCs	CRISPR/Cas9	*UBE3A* KO (5bp deletion on exon 6 causing a frameshift and early translational termination)
Sirois et al. (2020)	H9 ESC	CRISPR/Cas9 in the presence of ssODN	*UBE3A* Iso1 KO, Iso2 KO, and Iso3 KO (by disruption of each TSS)

Besides iPSC models, genetically edited stem cell lines targeting the *UBE3A* gene have also been engineered (Table 2). A *UBE3A* KO cell line was generated from a male iPSC line through the insertion of 1 bp at the translational start site of the UBE3A protein isoform 1 in both maternal and paternal alleles (Fink et al., 2017). More recently, two ESC lines, the male H1, and the female H9, were edited at the *UBE3A* locus using a single guide RNA targeting exon 6 that led to a 5 base pair deletion in exon 6 of this gene, causing a frameshift and early translational termination in the best characterized H9 clone (Sun et al., 2019). Furthermore, isogenic H9 ESCs that specifically lack one of the three individual *UBE3A* protein isoforms were generated through mutation of their independent translational start sites using CRISPR/Cas9 and single-stranded oligodeoxynucleotides (ssODN) templates to discern the relative contribution of each isoform for the building up of the AS phenotype (Sirois et al., 2020). These CRISPR/Cas9-engineered ESC models targeting *UBE3A* as a whole or its individual isoforms are powerful models for studying the localization and function of UBE3A (Sirois et al., 2020). However, they lack *UBE3A* expression already in the stem cell state and do not recapitulate the developmental path leading to neuronal-specific loss of *UBE3A* expression due to imprinting, reproduced in iPSC models derived from AS individuals (Chamberlain et al., 2010; Stanurova et al., 2016). Therefore, this should be considered when comparing the results acquired using non-edited iPSCs *versus UBE3A*-edited iPSC/ESC models.

With the increased use of stem cell models, researchers have noticed that reprogramming and long-term *in vitro* culture lead to the accumulation of genetic and epigenetic defects (Bar et al., 2017; Halliwell et al., 2020; Bansal et al., 2021). This includes imprinting defects which occur mainly during the process of iPSC reprogramming (Nazor et al., 2012; Ma et al., 2014; Bar et al., 2017; Arez et al., 2022). This is particularly concerning when using stem cell models, especially iPSCs, to model imprinting disorders such as AS. Fortunately, methylation profiles at PWS-IC or *SNURF* TSS-DMR, are not prone to reprogramming-induced errors in contrast to other imprinted loci such as *PEG3*, *IGF2-H19*, and *DLK1-DIO3* regions (Nazor et al., 2012; Ma et al., 2014; Bar et al., 2017; Klobučar et al., 2020). Indeed, all published AS iPSC lines have been confirmed to preserve the original methylation pattern at PWS-IC following iPSC reprogramming (Table 1) and recapitulate neuron-specific imprinting of *UBE3A* upon neuronal differentiation (Chamberlain et al., 2010; Stanurova et al., 2016). Nonetheless, Pólvora-Brandão et al. (2018) have reported a loss of maternal methylation at the PWS-IC in one out of five non-AS iPSC clones, originating a cell line mimicking the imprinting defect typical of AS ID iPSCs (Pólvora-Brandão et al., 2018). Validation of the correct methylation pattern at the PWS-IC is therefore mandatory to ensure that imprinting has not been lost in PSC models of AS and their controls.

Although a reasonable number of stem cell lines covering the major causes of the disease have been created to model AS, these cellular models have their own limitations. First, their number can be considered low, especially for AS UPD and ID cell lines, with only one of each having been generated (Takahashi et al., 2017; Neureiter et al., 2018). Second, by coincidence, there is a sex bias trend with fewer male than female AS iPSC lines (Table 1). Third, most of these lack appropriate controls such as CRISPR/Cas9 gene-corrected clones or familiar controls. Gene-corrected isogenic controls are ideal but in the context of AS are almost only applicable for *UBE3A* Mut cases. AS MD and AS IC could, in theory, also be recreated using non-AS iPSCs/ESCs through genetic or epigenetic editing (Qian et al., 2023; Zhou et al., 2023), respectively. This is not an option for AS UPD cases, where the genetic error (inheritance of two paternal chr15s) cannot be rescued with current editing techniques. In this case, the best available option to act as a control iPSC would be from non-affected parents or siblings. All in all, these points highlight the importance of enlarging the current portfolio of stem cell lines available for AS research, with an unbiased representation of both biological sexes and with appropriate genetically matched controls. These will guarantee the accuracy and reproducibility of the results gathered using AS stem cell models.

## Advances in Angelman syndrome research using human pluripotent stem cells

**FIGURE 4:**
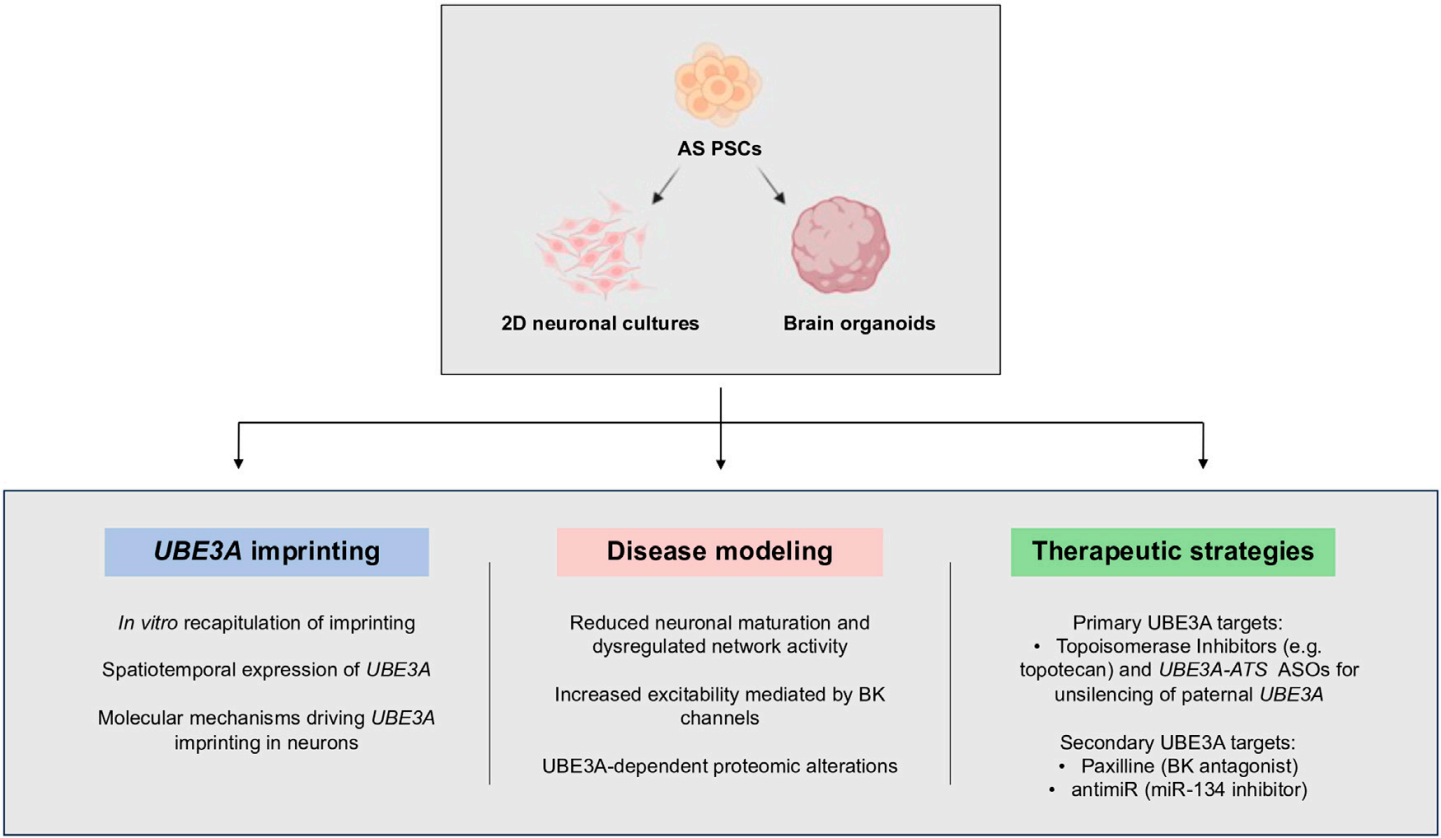
Applications of human pluripotent stem cells (PSCs) in Angelman syndrome research. Neuronal differentiation of iPSCs in 2D neuronal cultures and brain organoids has allowed the study of *UBE3A* imprinting *in vitro*, to discover neuronal phenotypes of the disease, and evaluate the potential of several therapeutic strategies. Abbreviations: BK—big potassium channels; ASOs—antisense oligonucleotides. PSCs—pluripotent stem cells. Created with Biorender.com.

Stem-cell-based research on AS is still in its infancy but has already led to innovative studies that increased our knowledge about this syndrome. To model AS, iPSCs/ESCs are usually submitted to neuronal differentiation either as a simple monolayer culture or more complex self-organized organ-like structures known as brain organoids (Pașca et al., 2022). Organoids have been defined as “*in vitro*-*generated cellular systems that emerge by self-organization, include multiple cell types, and exhibit some cytoarchitectural and functional features reminiscent of an organ or organ region*” (Pașca et al., 2022). Several protocols, relying mostly on intrinsic factors and spontaneous differentiation (unguided) or with controlled addition of external factors to direct the differentiation process (guided) can be followed to generate whole or region-specific brain organoids. Both 2D and 3D differentiation protocols resort to specific media and supplements/specific pathways inhibitors, to promote the development of neural progenitors and at later stages of differentiation, mature neurons and astrocytes aiming to recreate *in vitro* the developmental path of brain regions [reviewed in Paşca (2019)]. In particular, brain organoids have helped to overcome one of the biggest challenges associated with investigating neurological disorders, the difficulty of studying the human brain. While post-mortem samples and medical imaging techniques were and still are a source of information, they can only monitor disease progression and cannot track pathological processes at the cellular level (Eichmüller and Knoblich, 2022). A great part of AS research has also used mouse models to study the disease, although there are limitations here as well. The mouse brain differs from the human in key aspects such as size, architecture, and gyrification of the cortex. Furthermore, the regulation of some conserved pathways is different, leading to morphological, architectural, and connectivity differences between species (Eichmüller and Knoblich, 2022). Brain organoids fill the gap between these different models, allowing the study of neurodevelopmental disorders from early stages, at cellular and molecular levels, using human cells. In the case of AS, stem cell models have been used in three main contexts: 1) to understand the process of imprinting regulation at the chr15q11-q13; 2) as a disease model to find molecular/functional signatures of the disease; 3) for preclinical development of existing and novel therapeutic agents for future treatment for AS (Figure 4).

Several studies have been conducted in stem cells to unveil when and how *UBE3A* imprinting arises during *in vitro* differentiation. On one hand, these cell models provide an accessible system to study imprinting establishment. On the other hand, *UBE3A* imprinting is a prerequisite to validate their applicability for studying AS. *UBE3A* imprinting has been confirmed to occur during *in vitro* differentiation of AS iPSCs in both 2D cultures and organoids (Chamberlain et al., 2010; Stanurova et al., 2016; Sen et al., 2020). This has been perceived by the downregulation of *UBE3A* RNA and protein levels with a concomitant upregulation of *UBE3A-ATS* when the first neurons emerge in the culture. Formal proof of loss of the paternal *UBE3A* allele was obtained by Stanurova et al. (2016), who determined allelic ratios between the maternal and paternal alleles thanks to genetic variation caused by a missense 3 bp deletion in *UBE3A* gene in an AS Mut iPSC line. These studies pointed to a correlation between *UBE3A-ATS* expression and paternal *UBE3A* silencing and proved that iPSC-derived neuronal differentiation recapitulates *UBE3A* imprinting and can be an adequate model to study AS.

The group of S. Chamberlain has pioneered the use of engineered stem cell models to study the molecular mechanisms regulating imprinting at the chr15q11-q13 region (Chamberlain et al., 2010; Martins-Taylor et al., 2014; Hsiao et al., 2019). In one of their studies, they investigated the reasons explaining why *UBE3A* imprinting is restricted to neurons. Through a series of CRISPR/Cas9-induced deletions and inversions in iPSCs, they identified a boundary element around *IPW* and *PWAR1* genes that is responsible for terminating *SNHG14* transcription in non-neuronal cells. Ablation of such a genomic element enables transcription to extend beyond this boundary and leads to an earlier onset of paternal *UBE3A* silencing during neuronal differentiation (Hsiao et al., 2019). This study illustrates the capacity of genetic manipulation of stem cells to gain mechanistic insights into the regulation of neuronal-specific imprinting expression of *UBE3A*.

Neuronal differentiation of both iPSC and ESC has provided important insights into the spatiotemporal expression pattern of *UBE3A* (Sen et al., 2020), as well as on differences in location and neuronal function of several human UBE3A isoforms (Sirois et al., 2020). In H9 ESC-derived whole-brain organoids, nuclear UBE3A increases with the progress of differentiation, with a decreasing ratio of cytoplasmic to nuclear UBE3A. The protein appeared to be predominantly nuclear in neurons after only 3 weeks in culture, with this location increasing over time (Sen et al., 2020), matching previous results in mice (Judson et al., 2014). In another study, monitoring isogenic mutated ESC lines throughout neuronal differentiation further revealed that UBE3A isoform 1 predominates in both undifferentiated cells and neurons (Sirois et al., 2020). Loss of isoform 1 led to a significant reduction in total and cytoplasmic UBE3A levels (but not nuclear), while loss of isoforms 2 and 3 did not induce significant alterations in UBE3A levels. By Western blot, UBE3A was found to localize predominantly in the cytoplasm, in both ESC and neurons in this study. However, by immunofluorescence, the strongest UBE3A signal in neurons appears to be in the nucleus, although there was also a signal detected in neurites and the soma, outside the nucleus (Sirois et al., 2020). Future experiments are needed to gain insights into these apparently contradictory findings.

Elegant experiments using stem cell models of AS uncovered novel molecular/functional signatures of the disease. In contrast to the absence of significant morphological and functional changes at early differentiation stages, both in 2D (Fink et al., 2017) and 3D models (Sun et al., 2019), important alterations were observed at later time points, coinciding with the appearance of functionally mature neurons in culture (Fink et al., 2017; Sun et al., 2019). The first study that extensively characterized AS phenotype in long-term neuronal cultures (over 20 weeks) was developed by Fink et al. (2017), using AS iPSC-derived forebrain neurons. The authors used three AS-iPSC lines (two AS MD and one AS Mut), and an engineered *UBE3A* KO iPSC line. AS and control cultures presented similar cell composition throughout differentiation, with no significant differences between the proportion of glutamatergic and GABAergic neurons, astrocytes, or between upper and deep cortical layer markers. Nonetheless, alterations in neuronal excitability, functionality, and synaptic plasticity were detected, in line with previous research in mice models (Mardirossian et al., 2009; Kaphzan et al., 2011; Campbell et al., 2022; O’Geen et al., 2023). These effects included more depolarized resting membrane potentials (RMPs), a lower proportion of cells firing action potential (AP) spike trains, more immature APs, and fewer calcium transients. These effects were also proven to be UBE3A-dependent, as they were observed in *UBE3A* KO lines or when control iPSCs were treated with *UBE3A*-targeting ASOs to knockdown *UBE3A* expression (Fink et al., 2017). More precisely, isoform 1 is thought to be the most related to the observed neuronal phenotypes, as a KO line showed similar RMP depolarization (Sirois et al., 2020). Overall, these results point to a reduced developmental maturation, as well as dysregulated network activity and excitability features in AS, and prove the usefulness of AS stem cell models to find functional phenotypes and readouts of the disease.

Sun et al. (2019) developed the first study where two pairs of *UBE3A* KO hESCs and an AS MD iPSC line were differentiated not only in 2D but also in 3D, into cortical organoids. They uncovered a channelopathy mediated by an increase in BK channel density leading to increased neuronal excitability. This increase was due to the lack of UBE3A-mediated ubiquitination and proteasomal degradation of BK channels, as confirmed by *in vitro* and *in vivo* ubiquitination assays. Therefore, BK channels are a substrate of UBE3A and a putative therapeutic target of AS. All in all, this study has contributed to a better understanding of the mechanisms underlying network hyperactivity and epilepsy susceptibility in AS patients (Sun et al., 2019).

In another study, Pandya et al. (2021) took advantage of AS iPSC-derived neurons to perform a proteomic study and identify proteins whose abundance was responsive to changes in UBE3A levels. After an initial comparison between AS and control iPSC-derived neurons, the authors modulated the expression of *UBE3A* using ASOs against *UBE3A* (to reduce expression) or against *UBE3-ATS* (to increase expression) and repeated their proteomic workflow. A range from 70 to 225 proteins were identified with the main outcome being the discovery of the secreted retrovirus-like GAG-domain-containing protein PEG10 and associated proteins as being upregulated in AS neurons (Pandya et al., 2021). This was also confirmed in post-mortem brain samples of AS individuals. Curiously, this was not observed in mice, which could suggest a relevant mechanistic difference between AS humans and AS mouse models. Although PEG10 was found not to be a ubiquitin target of UBE3A, it was still targeted to the proteasome in a UBE3A-dependent manner. Interestingly, PEG10 downregulation in AS iPSC-derived neurons results in a transcriptomic response similar to what happens upon UBE3A reinstatement, suggesting that PEG10 could contribute to the pathophysiology of AS (Pandya et al., 2021). Interestingly, PEG10, also encoded by a paternally imprinted gene, is recruited to stress granules where it interacts with several RNAs and is secreted in extracellular vesicles. How this could be related to its putative role in AS pathogenesis still needs to be further explored. As a secreted protein, PEG10 is also a promising biomarker to consider for AS therapeutics currently advancing in clinical trials.

Stem cell models of AS are also becoming key for the testing and development of potential new therapeutic strategies. They have been used for either 1) testing inhibitors of UBE3A targets or 2) using molecules targeting the molecular cause of the disease. To exemplify the first case, Sun et al. (2019) tested paxilline, a BK blocker, after they discovered that these channels were increased in AS iPSC-derived neurons. They showed that paxilline was able to revert altered excitability and abnormal AP firing in both AS 2D neuronal cultures and cortical organoids. This finding was further supported by the amelioration of seizure threshold and susceptibility when administered in a mouse model of AS (Sun et al., 2019). These results position BK channels as promising therapeutic targets for the treatment of seizures in AS individuals (Sun et al., 2019). Another interesting study explored the idea that *UBE3A* mRNA might function as a sponge for the microRNA miR-134 (Campbell et al., 2022). An antimiR oligonucleotide inhibitor of miR-134 was shown to upregulate its targets in neurons differentiated from AS MD iPSCs and ameliorate AS phenotypes in *UBE3A* ^*mat-/pat+*^ mice upon intracerebroventricular injection. These findings give the prospect that microRNA modulation could be beneficial in treating some clinically relevant symptoms affecting AS individuals.

The most widely investigated therapeutic option is the reinstatement of UBE3A through unsilencing of the paternal copy of the gene. This is achieved by disrupting the *UBE3A-ATS* RNA and can be achieved in multiple ways (Huang et al., 2012; Meng et al., 2015; Wolter et al., 2020; Schmid et al., 2021; Dindot et al., 2023; Li et al., 2023; O’Geen et al., 2023). One such way uses topoisomerase inhibitors, such as topotecan or indotecan (Huang et al., 2012; Lee et al., 2018). Administration of 1 µM topotecan to *in vitro* iPSC-derived neuronal cultures led to a ∼50% increase in *UBE3A* mRNA expression and rescued abnormal AP firing, RMP depolarization, and synaptic frequency (Fink et al., 2017). Administration of 1 µM topotecan or indotecan to AS cerebral organoids had similar effects, resulting in a knockdown of *UBE3A-ATS* and increased *UBE3A* in neurons, with a single dose of indotecan being able to persistently rescue *UBE3A* for 10-to-17 days after exposure. As a result, calcium transient phenotypes in AS organoids were also reverted (Sen et al., 2020). The problem with topoisomerase inhibitors is that they disrupt the full transcriptional unit that contains *SNURF/SNRPN* and *snoRNA* genes which are involved in PWS, besides causing the downregulation of several long RNA transcripts (Huang et al., 2012; King et al., 2013; Tan and Bird, 2016; Lee et al., 2018). A preferential strategy will hit specifically the *UBE3A-ATS* gene only.

Targeting the *UBE3A-ATS* specifically using modified ASOs was found to successfully unsilence paternal *UBE3A* without disrupting the expression of the other transcripts implicated in PWS and rescue several disease phenotypes in the AS mouse model (Meng et al., 2015). This proof-of-principle experiment showed the unprecedented ability of this approach to target the molecular cause of AS. However, the efficient ASOs found in mice hold no homology in the human genome, therefore, human sequence-specific ASOs needed to be tested in human cells. The optimal cellular system for this purpose would be human ESC/iPSC-derived neurons. An additional advantage is that iPSCs can be derived from individuals with different molecular causes of the disease, enabling the monitoring of ASO efficacy in distinct (epi)genetic backgrounds of the disease. An earlier publication showed that human-specific ASOs were able to downregulate *UBE3A-ATS* and activate *UBE3A* in iPSC-derived neurons (Pandya et al., 2021). More recently, Dindot et al. (2023) have perfected ASO chemistry to target an evolutionary conserved region at the start of *UBE3A-ATS* to efficiently repress *UBE3-ATS* transcription and reactivate paternal *UBE3A*. After this screening study in iPSC-derived neurons, the best ASO candidates were administered by lumbar intrathecal injections in cynomolgus monkeys, and promising results were obtained with minor adverse effects (Dindot et al., 2023). These findings drove the first molecular therapy for AS to go into clinical development in phase I/II (ClinicalTrials.gov, NCT04259281). A similar trial from Roche using the same technology also reached phase I clinical trials (ClinicalTrials.gov, NCT04428281). This is illustrative of the power of stem cell-based research to study diseases such as AS.

## Conclusion remarks and future perspectives

In this article, we provide a comprehensive overview of stem cell-based research in AS and contextualize it within the historical advancements made over the past ∼50/60 years in understanding this disease. Although the use of human stem cell models of AS is relatively recent, it already provided significant scientific progress in uncovering novel pathophysiological aspects of the disorder, as well as in identifying new proteins affected by UBE3A that could be targets for future therapeutic interventions. Additionally, stem cell models are serving as valuable preclinical tools for evaluating human sequence-specific genetic therapies such as ASOs or CRISPR-based gene editing. In the forthcoming years, the AS stem cell model portfolio will likely expand, hopefully encompassing the full spectrum of (epi)genetic variability observed in this condition. Current concerns in the epigenetic and genetic fidelity of human stem cells are being tackled (Pham et al., 2022) and hopefully will be solved in the near future. Also, there are grand expectations for further development of stem cell-based disease modeling with the potential to revolutionize biomedical research, drug development, and, ultimately, patient care. More complex multi-organ models such as assembloids or organ-on-chip systems will provide a more comprehensive understanding of disease pathogenesis, and also a better prediction of drug responses in the human body (Zhang et al., 2018; Miura et al., 2022; Tenreiro et al., 2023). Improvement of long-term organoid culture by optimizing dynamic culture methods and incorporating supportive cell types and biomaterials would also aid in the study of disease pathology providing a deeper insight into the mechanisms underlying disease development (Giandomenico et al., 2021). Advances are also being made in the automation and miniaturization of stem cell-based assays to improve high-throughput screening and accelerate drug discovery processes (Brandenberg et al., 2020). The constant refinement in gene editing technologies (Anzalone et al., 2020) as well as the production of single-cell and spatial transcriptomics data (Vandereyken et al., 2023) would also produce novel cellular models and datasets that would accelerate our collective understanding of human diseases, such as AS. In conclusion, stem cell-based models hold immense promise to revolutionize the lives of AS patients by paving the way toward transformative treatment addressing the root causes of the disease, and offering hope for improved cognitive, motor, and behavioral outcomes.

## References

[B1] Aguilera C. Gabau E. Ramirez-Mallafré A. Brun-Gasca C. Dominguez-Carral J. Delgadillo V. (2021). New genes involved in Angelman syndrome-like: expanding the genetic spectrum. PLOS ONE 16 (10), e0258766. 10.1371/journal.pone.0258766 34653234 PMC8519432

[B2] Albrecht U. Sutcliffe J. S. Cattanach B. M. Beechey C. V. Armstrong D. Eichele G. (1997). Imprinted expression of the murine Angelman syndrome gene, Ube3a, in hippocampal and Purkinje neurons. Nat. Genet. 17 (1), 75–78. 10.1038/ng0997-75 9288101

[B3] Angelman H. (1965). Puppet children A report on three cases. Dev. Med. Child Neurology 7 (6), 681–688. 10.1111/j.1469-8749.1965.tb07844.x 18754889

[B4] Anitha A. Thanseem I. Iype M. Thomas S. V. (2023). Mitochondrial dysfunction in cognitive neurodevelopmental disorders: cause or effect? Mitochondrion 69, 18–32. 10.1016/j.mito.2023.01.002 36621534

[B5] Anzalone A. V. Koblan L. W. Liu D. R. (2020). Genome editing with CRISPR–Cas nucleases, base editors, transposases and prime editors. Nat. Biotechnol. 38 (7), 824–844. 10.1038/s41587-020-0561-9 32572269

[B6] Arez M. Eckersley-Maslin M. Klobučar T. von Gilsa Lopes J. Krueger F. Mupo A. (2022). Imprinting fidelity in mouse iPSCs depends on sex of donor cell and medium formulation. Nat. Commun. 13 (1), 5432. 10.1038/s41467-022-33013-5 36114205 PMC9481624

[B7] Avagliano Trezza R. Punt A. M. Mientjes E. van den Berg M. Zampeta F. I. de Graaf I. J. (2021). Mono-ubiquitination of Rabphilin 3A by UBE3A serves a non-degradative function. Sci. Rep. 11 (1), 3007. 10.1038/s41598-021-82319-9 33542309 PMC7862399

[B8] Avagliano Trezza R. Sonzogni M. Bossuyt S. N. V. Zampeta F. I. Punt A. M. van den Berg M. (2019). Loss of nuclear UBE3A causes electrophysiological and behavioral deficits in mice and is associated with Angelman syndrome. Nat. Neurosci. 22 (8), 1235–1247. 10.1038/s41593-019-0425-0 31235931

[B9] Bansal P. Ahern D. T. Kondaveeti Y. Qiu C. W. Pinter S. F. (2021). Contiguous erosion of the inactive X in human pluripotency concludes with global DNA hypomethylation. Cell. Rep. 35 (10), 109215. 10.1016/j.celrep.2021.109215 34107261 PMC8267460

[B10] Bar S. Schachter M. Eldar-Geva T. Benvenisty N. (2017). Large-Scale analysis of loss of imprinting in human pluripotent stem cells. Cell. Rep. 19 (5), 957–968. 10.1016/j.celrep.2017.04.020 28467909

[B11] Beasley S. A. Kellum C. E. Orlomoski R. J. Idrizi F. Spratt D. E. (2020). An Angelman syndrome substitution in the HECT E3 ubiquitin ligase C-terminal Lobe of E6AP affects protein stability and activity. PLOS ONE 15 (7), e0235925. 10.1371/journal.pone.0235925 32639967 PMC7343168

[B12] Berg E. L. Pride M. C. Petkova S. P. Lee R. D. Copping N. A. Shen Y. (2020). Translational outcomes in a full gene deletion of ubiquitin protein ligase E3A rat model of Angelman syndrome. Transl. Psychiatry 10 (1), 39. 10.1038/s41398-020-0720-2 32066685 PMC7026078

[B13] Berg J. M. Pakula Z. (1972). Angelman’s (“Happy puppet”) syndrome. Archives Pediatr. Adolesc. Med. 123 (1), 72–74. 10.1001/archpedi.1972.02110070122020 5010558

[B14] Berkowitz B. A. Lenning J. Khetarpal N. Tran C. Wu J. Y. Berri A. M. (2017). *In vivo* imaging of prodromal hippocampus CA1 subfield oxidative stress in models of Alzheimer disease and Angelman syndrome. FASEB J. 31 (9), 4179–4186. 10.1096/fj.201700229R 28592637 PMC6207301

[B15] Beygo J. Grosser C. Kaya S. Mertel C. Buiting K. Horsthemke B. (2020). Common genetic variation in the Angelman syndrome imprinting centre affects the imprinting of chromosome 15. Eur. J. Hum. Genet. 28 (6), 835–839. 10.1038/s41431-020-0595-y 32152487 PMC7253442

[B16] Bird L. (2014). Angelman syndrome: review of clinical and molecular aspects. Appl. Clin. Genet. 7, 93–104. 10.2147/TACG.S57386 24876791 PMC4036146

[B17] Born H. A. Martinez L. A. Levine A. T. Harris S. E. Mehra S. Lee W. L. (2021). Early developmental EEG and seizure phenotypes in a full gene deletion of ubiquitin protein ligase E3A rat model of angelman syndrome. eneuro 8 (2), ENEURO.0345–20.2020. 10.1523/ENEURO.0345-20.2020 33531368 PMC8114899

[B18] Bossuyt S. N. V. Punt A. M. de Graaf I. J. van den Burg J. Williams M. G. Heussler H. (2021). Loss of nuclear UBE3A activity is the predominant cause of Angelman syndrome in individuals carrying UBE3A missense mutations. Hum. Mol. Genet. 30 (6), 430–442. 10.1093/hmg/ddab050 33607653 PMC8101352

[B19] Bower B. D. Jeavons P. M. (1967). The “happy puppet” syndrome. Archives Dis. Child. 42 (223), 298–302. 10.1136/adc.42.223.298 PMC2019739 6025370

[B20] Brandenberg N. Hoehnel S. Kuttler F. Homicsko K. Ceroni C. Ringel T. (2020). High-throughput automated organoid culture via stem-cell aggregation in microcavity arrays. Nat. Biomed. Eng. 4 (9), 863–874. 10.1038/s41551-020-0565-2 32514094

[B21] Brennan M. L. Adam M. P. Seaver L. H. Myers A. Schelley S. Zadeh N. (2015). Increased body mass in infancy and early toddlerhood in Angelman syndrome patients with uniparental disomy and imprinting center defects. Am. J. Med. Genet. Part A 167 (1), 142–146. 10.1002/ajmg.a.36831 25402239

[B22] Bruinsma C. F. Schonewille M. Gao Z. Aronica E. M. A. Judson M. C. Philpot B. D. (2015). Dissociation of locomotor and cerebellar deficits in a murine Angelman syndrome model. J. Clin. Investigation 125 (11), 4305–4315. 10.1172/JCI83541 PMC4639977 26485287

[B23] Buiting K. Saitoh S. Gross S. Dittrich B. Schwartz S. Nicholls R. D. (1995). Inherited microdeletions in the Angelman and Prader–Willi syndromes define an imprinting centre on human chromosome 15. Nat. Genet. 9 (4), 395–400. 10.1038/ng0495-395 7795645

[B24] Buiting K. Williams C. Horsthemke B. (2016). Angelman syndrome — insights into a rare neurogenetic disorder. Nat. Rev. Neurol. 12 (10), 584–593. 10.1038/nrneurol.2016.133 27615419

[B25] Burnside R. D. Pasion R. Mikhail F. M. Carroll A. J. Robin N. H. Youngs E. L. (2011). Microdeletion/microduplication of proximal 15q11.2 between BP1 and BP2: a susceptibility region for neurological dysfunction including developmental and language delay. Hum. Genet. 130 (4), 517–528. 10.1007/s00439-011-0970-4 21359847 PMC6814187

[B26] Butler M. Palmer C. G. (1983). Parental origin of chromosome 15 deletion in Prader-Willi Syndrome. Lancet 321 (8336), 1285–1286. 10.1016/S0140-6736(83)92745-9 PMC5510872 6134086

[B27] Campbell A. Morris G. Sanfeliu A. Augusto J. Langa E. Kesavan J. C. (2022). AntimiR targeting of microRNA-134 reduces seizures in a mouse model of Angelman syndrome. Mol. Ther. - Nucleic Acids 28, 514–529. 10.1016/j.omtn.2022.04.009 35592499 PMC9092865

[B28] Cattanach B. M. Barr J. A. Beechey C. V. Martin J. Noebels J. Jones J. (1997). A candidate model for angelman syndrome in the mouse. Mamm. Genome 8 (7), 472–478. 10.1007/s003359900479 9195990

[B29] Chamberlain S. J. Brannan C. I. (2001). The prader–willi syndrome imprinting center activates the paternally expressed murine Ube3a antisense transcript but represses paternal Ube3a. Genomics 73 (3), 316–322. 10.1006/geno.2001.6543 11350123

[B30] Chamberlain S. J. Chen P. F. Ng K. Y. Bourgois-Rocha F. Lemtiri-Chlieh F. Levine E. S. (2010). Induced pluripotent stem cell models of the genomic imprinting disorders Angelman and Prader-Willi syndromes. Proc. Natl. Acad. Sci. U. S. A. 107 (41), 17668–17673. 10.1073/pnas.1004487107 20876107 PMC2955112

[B31] Clayton-Smith J. Driscoll D. J. Waters M. F. Webb T. Andrews T. Malcolm S. (1993). Difference in methylation patterns within the D15S9 region of chromosome 15q11-13 in first cousins with Angelman syndrome and Prader-Willi syndrome. Am. J. Med. Genet. 47 (5), 683–686. 10.1002/ajmg.1320470519 8266996

[B32] Colas D. Wagstaff J. Fort P. Salvert D. Sarda N. (2005). Sleep disturbances in Ube3a maternal-deficient mice modeling Angelman syndrome. Neurobiol. Dis. 20 (2), 471–478. 10.1016/j.nbd.2005.04.003 15921919

[B33] Copping N. A. Christian S. G. B. Ritter D. J. Islam M. S. Buscher N. Zolkowska D. (2017). Neuronal overexpression of Ube3a isoform 2 causes behavioral impairments and neuroanatomical pathology relevant to 15q11.2-q13.3 duplication syndrome. Hum. Mol. Genet. 26 (20), 3995–4010. 10.1093/hmg/ddx289 29016856 PMC5886211

[B34] Costa-Mattioli M. Monteggia L. M. (2013). mTOR complexes in neurodevelopmental and neuropsychiatric disorders. Nat. Neurosci. 16 (11), 1537–1543. 10.1038/nn.3546 24165680

[B35] Cubillos-Rojas M. Schneider T. Hadjebi O. Pedrazza L. de Oliveira J. R. Langa F. (2016). The HERC2 ubiquitin ligase is essential for embryonic development and regulates motor coordination. Oncotarget 7 (35), 56083–56106. 10.18632/oncotarget.11270 27528230 PMC5302898

[B36] da Rocha S. T. Gendrel A. V. (2019). The influence of DNA methylation on monoallelic expression. Essays Biochem. 63 (6), 663–676. 10.1042/EBC20190034 31782494 PMC6923323

[B37] Daily J. L. Nash K. Jinwal U. Golde T. Rogers J. Peters M. M. (2011). Adeno-associated virus-mediated rescue of the cognitive defects in a mouse model for angelman syndrome. PLoS ONE 6 (12), e27221. 10.1371/journal.pone.0027221 22174738 PMC3235088

[B38] Damgaard R. B. (2021). The ubiquitin system: from cell signalling to disease biology and new therapeutic opportunities. Cell. Death Differ. 28 (2), 423–426. 10.1038/s41418-020-00703-w 33446876 PMC7862391

[B39] Delahanty R. J. Zhang Y. Bichell T. J. Shen W. Verdier K. Macdonald R. L. (2016). Beyond epilepsy and autism: disruption of GABRB3 causes ocular hypopigmentation. Cell. Rep. 17 (12), 3115–3124. 10.1016/j.celrep.2016.11.067 28009282 PMC5240804

[B40] Dickinson A. J. Fielder A. R. Young I. D. Duckett D. P. (1990). Ocular findings in angelman’s (happy puppet) syndrome. Ophthalmic Paediatr. Genet. 11 (1), 1–6. 10.3109/13816819009012942 2348977

[B41] Dindot S. V. Antalffy B. A. Bhattacharjee M. B. Beaudet A. L. (2007). The Angelman syndrome ubiquitin ligase localizes to the synapse and nucleus, and maternal deficiency results in abnormal dendritic spine morphology. Hum. Mol. Genet. 17 (1), 111–118. 10.1093/hmg/ddm288 17940072

[B42] Dindot S. V. Christian S. Murphy W. J. Berent A. Panagoulias J. Schlafer A. (2023). An ASO therapy for Angelman syndrome that targets an evolutionarily conserved region at the start of the UBE3A-AS transcript. Sci. Transl. Med. 15 (688), eabf4077. 10.1126/scitranslmed.abf4077 36947593

[B43] Dittrich B. Robinson W. P. Knoblauch H. Buiting K. Schmidt K. Gillessen-Kaesbach G. (1992). Molecular diagnosis of the Prader-Willi and Angelman syndromes by detection of parent-of-origin specific DNA methylation in 15q11-13. Hum. Genet. 90 (3), 313–315. 10.1007/BF00220089 1487250

[B44] Dodge A. Peters M. M. Greene H. E. Dietrick C. Botelho R. Chung D. (2020). Generation of a novel rat model of angelman syndrome with a complete *Ube3a* gene deletion. Autism Res. 13 (3), 397–409. 10.1002/aur.2267 31961493 PMC7787396

[B45] Driscoll D. J. Waters M. F. Williams C. A. Zori R. T. Glenn C. C. Avidano K. M. (1992). A DNA methylation imprint, determined by the sex of the parent, distinguishes the angelman and Prader-Willi syndromes. Genomics 13 (4), 917–924. 10.1016/0888-7543(92)90001-9 1505981

[B46] Egawa K. Kitagawa K. Inoue K. Takayama M. Takayama C. Saitoh S. (2012). Decreased tonic inhibition in cerebellar granule cells causes motor dysfunction in a mouse model of angelman syndrome. Sci. Transl. Med. 4 (163), 163ra157. 10.1126/scitranslmed.3004655 23220633

[B47] Eichmüller O. L. Knoblich J. A. (2022). Human cerebral organoids — a new tool for clinical neurology research. Nat. Rev. Neurol. 18 (11), 661–680. 10.1038/s41582-022-00723-9 36253568 PMC9576133

[B48] Fink J. J. Robinson T. M. Germain N. D. Sirois C. L. Bolduc K. A. Ward A. J. (2017). Disrupted neuronal maturation in Angelman syndrome-derived induced pluripotent stem cells. Nat. Commun. 8, 15038. 10.1038/ncomms15038 28436452 PMC5413969

[B49] Galligan J. T. Martinez-Noël G. Arndt V. Hayes S. Chittenden T. W. Harper J. W. (2015). Proteomic analysis and identification of cellular interactors of the giant ubiquitin ligase HERC2. J. Proteome Res. 14 (2), 953–966. 10.1021/pr501005v 25476789 PMC4324439

[B50] Gentile J. K. Tan W. H. Horowitz L. T. Bacino C. A. Skinner S. A. Barbieri-Welge R. (2010). A neurodevelopmental survey of angelman syndrome with genotype-phenotype correlations. J. Dev. Behav. Pediatr. 31 (7), 592–601. 10.1097/DBP.0b013e3181ee408e 20729760 PMC2997715

[B51] Giandomenico S. L. Sutcliffe M. Lancaster M. A. (2021). Generation and long-term culture of advanced cerebral organoids for studying later stages of neural development. Nat. Protoc. 16 (2), 579–602. 10.1038/s41596-020-00433-w 33328611 PMC7611064

[B52] Greer P. L. Hanayama R. Bloodgood B. L. Mardinly A. R. Lipton D. M. Flavell S. W. (2010). The angelman syndrome protein Ube3A regulates synapse development by ubiquitinating arc. Cell. 140 (5), 704–716. 10.1016/j.cell.2010.01.026 20211139 PMC2843143

[B53] Gu B. Carstens K. E. Judson M. C. Dalton K. A. Rougié M. Clark E. P. (2018). Ube3a reinstatement mitigates epileptogenesis in Angelman syndrome model mice. J. Clin. Investigation 129 (1), 163–168. 10.1172/JCI120816 PMC6307939 30352049

[B54] Hall J. G. (1990). Angelman’s syndrome, abnormality of 15q11-13, and imprinting. J. Med. Genet. 27 (2), 141. 10.1136/jmg.27.2.141 PMC1016940 2319586

[B55] Halliwell J. Barbaric I. Andrews P. W. (2020). Acquired genetic changes in human pluripotent stem cells: origins and consequences. Nat. Rev. Mol. Cell. Biol. 21 (12), 715–728. 10.1038/s41580-020-00292-z 32968234

[B56] Horsthemke B. Buiting K. (2006). Imprinting defects on human chromosome 15. Cytogenet. Genome Res. 113 (1–4), 292–299. 10.1159/000090844 16575192

[B57] Horsthemke B. Wagstaff J. (2008). Mechanisms of imprinting of the Prader-Willi/Angelman region. Am. J. Med. Genet. Part A 146A (16), 2041–2052. 10.1002/ajmg.a.32364 18627066

[B58] Hsiao J. S. Germain N. D. Wilderman A. Stoddard C. Wojenski L. A. Villafano G. J. (2019). A bipartite boundary element restricts *UBE3A* imprinting to mature neurons. Proc. Natl. Acad. Sci. 116 (6), 2181–2186. 10.1073/pnas.1815279116 30674673 PMC6369781

[B59] Huang H. S. Allen J. A. Mabb A. M. King I. F. Miriyala J. Taylor-Blake B. (2012). Topoisomerase inhibitors unsilence the dormant allele of Ube3a in neurons. Nature 481 (7380), 185–189. 10.1038/nature10726 PMC3257422 22190039

[B60] Huibregtse J. M. Scheffner M. Howley P. M. (1993). Cloning and expression of the cDNA for E6-AP, a protein that mediates the interaction of the human papillomavirus E6 oncoprotein with p53. Mol. Cell. Biol. 13 (2), 775–784. 10.1128/mcb.13.2.775 8380895 PMC358960

[B61] Jay V. Becker L. E. Chan F. W. Perry T. L. (1991). Puppet-like syndrome of Angelman: a pathologic and neurochemical study. Neurology 41 (3), 416–422. 10.1212/WNL.41.3.416 2006012

[B62] Jiang Y. Armstrong D. Albrecht U. Atkins C. M. Noebels J. L. Eichele G. (1998). Mutation of the angelman ubiquitin ligase in mice causes increased cytoplasmic p53 and deficits of contextual learning and long-term potentiation. Neuron 21 (4), 799–811. 10.1016/S0896-6273(00)80596-6 9808466

[B63] Jiang Y. Pan Y. Zhu L. Landa L. Yoo J. Spencer C. (2010). Altered ultrasonic vocalization and impaired learning and memory in angelman syndrome mouse model with a large maternal deletion from Ube3a to Gabrb3. PLoS ONE 5 (8), e12278. 10.1371/journal.pone.0012278 20808828 PMC2924885

[B64] Judson M. C. Sosa-Pagan J. O. Del Cid W. A. Han J. E. Philpot B. D. (2014). Allelic specificity of Ube3a expression in the mouse brain during postnatal development. J. Comp. Neurology 522 (8), 1874–1896. 10.1002/cne.23507 PMC3984624 24254964

[B65] Kaphzan H. Buffington S. A. Jung J. I. Rasband M. N. Klann E. (2011). Alterations in intrinsic membrane properties and the axon initial segment in a mouse model of angelman syndrome. J. Neurosci. 31 (48), 17637–17648. 10.1523/JNEUROSCI.4162-11.2011 22131424 PMC3483031

[B66] Kaplan L. C. Wharton R. Elias E. Mandell F. Donlon T. Latt S. A. (1987). Clinical heterogeneity associated with deletions in the long arm of chromosome 15: report of 3 new cases and their possible genetic significance. Am. J. Med. Genet. 28 (1), 45–53. 10.1002/ajmg.1320280107 3674117

[B67] Keating D. J. (2008). Mitochondrial dysfunction, oxidative stress, regulation of exocytosis and their relevance to neurodegenerative diseases. J. Neurochem. 104 (3), 298–305. 10.1111/j.1471-4159.2007.04997.x 17961149

[B68] Kelley K. W. Pasca S. P. (2022). Human brain organogenesis: toward a cellular understanding of development and disease. Cell. 185 (1), 42–61. 10.1016/j.cell.2021.10.003 34774127 PMC13523617

[B69] Keute M. Miller M. T. Krishnan M. L. Sadhwani A. Chamberlain S. Thibert R. L. (2021). Angelman syndrome genotypes manifest varying degrees of clinical severity and developmental impairment. Mol. Psychiatry 26 (7), 3625–3633. 10.1038/s41380-020-0858-6 32792659 PMC8505254

[B70] King I. F. Yandava C. N. Mabb A. M. Hsiao J. S. Huang H. S. Pearson B. L. (2013). Topoisomerases facilitate transcription of long genes linked to autism. Nature 501 (7465), 58–62. 10.1038/nature12504 23995680 PMC3767287

[B71] Kishino T. Lalande M. Wagstaff J. (1997). UBE3A/E6-AP mutations cause Angelman syndrome. Nat. Genet. 15 (1), 70–73. 10.1038/ng0197-70 8988171

[B72] Klobučar T. Kreibich E. Krueger F. Arez M. Pólvora-Brandão D. von Meyenn F. (2020). IMPLICON: an ultra-deep sequencing method to uncover DNA methylation at imprinted regions. Nucleic Acids Res. 48 (16), E92. 10.1093/nar/gkaa567 32621604 PMC7498334

[B73] Knoll J. H. M. Nicholls R. D. Magenis R. E. Graham J. M. Lalande M. Latt S. A. (1989). Angelman and Prader-Willi syndromes share a common chromosome 15 deletion but differ in parental origin of the deletion. Am. J. Med. Genet. 32 (2), 285–290. 10.1002/ajmg.1320320235 2564739

[B74] Kühnle S. Kogel U. Glockzin S. Marquardt A. Ciechanover A. Matentzoglu K. (2011). Physical and functional interaction of the HECT ubiquitin-protein ligases E6AP and HERC2. J. Biol. Chem. 286 (22), 19410–19416. 10.1074/jbc.M110.205211 21493713 PMC3103319

[B75] Lee H. M. Clark E. P. Kuijer M. B. Cushman M. Pommier Y. Philpot B. D. (2018). Characterization and structure-activity relationships of indenoisoquinoline-derived topoisomerase I inhibitors in unsilencing the dormant Ube3a gene associated with Angelman syndrome. Mol. Autism 9 (1), 45. 10.1186/s13229-018-0228-2 30140420 PMC6098585

[B76] Li E. Beard C. Jaenisch R. (1993). Role for DNA methylation in genomic imprinting. Nature 366 (6453), 362–365. 10.1038/366362a0 8247133

[B77] Li J. Shen Z. Liu Y. Yan Z. Liu Y. Lin X. (2023). A high-fidelity RNA-targeting Cas13 restores paternal Ube3a expression and improves motor functions in Angelman syndrome mice. Mol. Ther. 31 (7), 2286–2295. 10.1016/j.ymthe.2023.02.015 36805082 PMC10362381

[B78] Li S. Zhu Q. Cai Y. Yang Q. (2022). Generation of an induced pluripotent stem cell line from a patient with Angelman syndrome carrying UBE3A mutation. Stem Cell. Res. 62, 102791. 10.1016/j.scr.2022.102791 35489268

[B79] Liu G. Y. Sabatini D. M. (2020). mTOR at the nexus of nutrition, growth, ageing and disease. Nat. Rev. Mol. Cell. Biol. 21 (4), 183–203. 10.1038/s41580-019-0199-y 31937935 PMC7102936

[B80] Llewellyn K. J. Nalbandian A. Gomez A. Wei D. Walker N. Kimonis V. E. (2015). Administration of CoQ10 analogue ameliorates dysfunction of the mitochondrial respiratory chain in a mouse model of Angelman syndrome. Neurobiol. Dis. 76, 77–86. 10.1016/j.nbd.2015.01.005 25684537

[B81] Luk H. M. Lo I. F. M. (2016). Angelman syndrome in Hong Kong Chinese: a 20 years’ experience. Eur. J. Med. Genet. 59 (6–7), 315–319. 10.1016/j.ejmg.2016.05.003 27174604

[B82] Lusk L. Vogel-Farley V. DiStefano C. (2021). Maternal 15q duplication syndrome, GeneReviews®. Available at: https://www.ncbi.nlm.nih.gov/books/NBK367946/ (Accessed: August 1, 2023). 27308687

[B83] Ma H. Morey R. O'Neil R. C. He Y. Daughtry B. Schultz M. D. (2014). Abnormalities in human pluripotent cells due to reprogramming mechanisms. Nature 511 (7508), 177–183. 10.1038/nature13551 25008523 PMC4898064

[B84] Magenis R. E. Brown M. G. Lacy D. A. Budden S. LaFranchi S. (1987). Is angelman syndrome an alternate result of del(15)(qllql3)? Am. J. Med. Genet. 28 (4), 829–838. 10.1002/ajmg.1320280407 3688021

[B85] Malcolm S. Clayton-Smith J. Nichols M. Robb S. Webb T. Armour J. A. (1991). Uniparental paternal disomy in Angelman’s syndrome. Lancet 337 (8743), 694–697. 10.1016/0140-6736(91)90278-W 1672177

[B86] Maranga C. Fernandes T. G. Bekman E. da Rocha S. T. (2020). Angelman syndrome: a journey through the brain. FEBS J. 287 (11), 2154–2175. 10.1111/febs.15258 32087041

[B87] Maranga C. Pereira C. Raposo A. C. Vieira A. Duarte S. Bekman E. P. (2022). Generation and characterization of induced pluripotent stem cell line (IBBISTi004-A) from an Angelman syndrome patient carrying a class II deletion of the maternal chromosome 15q11.2-q13. Stem Cell. Res. 61, 102757. 10.1016/j.scr.2022.102757 35339881

[B88] Maranga C. Vieira A. A. Bekman E. P. da Rocha S. T. (2021). Induced pluripotent stem cells for modeling Angelman syndrome. iPSCs Model. Central Nerv. Syst. Disord. 6, 217–238. 10.1016/B978-0-323-85764-2.00015-6

[B89] Mardirossian S. Rampon C. Salvert D. Fort P. Sarda N. (2009). Impaired hippocampal plasticity and altered neurogenesis in adult Ube3a maternal deficient mouse model for Angelman syndrome. Exp. Neurol. 220 (2), 341–348. 10.1016/j.expneurol.2009.08.035 19782683

[B90] Margolis S. S. Sell G. L. Zbinden M. A. Bird L. M. (2015). Angelman syndrome. Neurotherapeutics 12 (3), 641–650. 10.1007/s13311-015-0361-y 26040994 PMC4489961

[B91] Martins-Taylor K. Hsiao J. S. Chen P. F. Glatt-Deeley H. De Smith A. J. Blakemore A. I. F. (2014). Imprinted expression of UBE3A in non-neuronal cells from a Prader–Willi syndrome patient with an atypical deletion. Hum. Mol. Genet. 23 (9), 2364–2373. 10.1093/hmg/ddt628 24363065 PMC3976333

[B92] Mathieu N. A. Levin R. H. Spratt D. E. (2021). Exploring the roles of HERC2 and the NEDD4L HECT E3 ubiquitin ligase subfamily in p53 signaling and the DNA damage response. Front. Oncol. 11, 659049. 10.3389/fonc.2021.659049 33869064 PMC8044464

[B93] Matsuura T. Sutcliffe J. S. Fang P. Galjaard R. J. Jiang Y. H. Benton C. S. (1997). *De novo* truncating mutations in E6-AP ubiquitin-protein ligase gene (UBE3A) in Angelman syndrome. Nat. Genet. 15 (1), 74–77. 10.1038/ng0197-74 8988172

[B94] Mayo Clinic (2022). Angelman syndrome. Available at: https://www.mayoclinic.org/diseases-conditions/angelman-syndrome/symptoms-causes/syc-20355621 (Accessed: July 7, 2023).

[B95] McKnight D. Bean L. Karbassi I. Beattie K. Bienvenu T. Bonin H. (2022). Recommendations by the ClinGen Rett/Angelman‐like expert panel for gene‐specific variant interpretation methods. Hum. Mutat. 43 (8), 1097–1113. 10.1002/humu.24302 34837432 PMC9135956

[B96] MedlinePlus (2022). UBE3A gene: medlinePlus genetics. Available at: https://medlineplus.gov/genetics/gene/ube3a/ (Accessed: July 6, 2023).

[B97] Medvar B. Raghuram V. Pisitkun T. Sarkar A. Knepper M. A. (2016). Comprehensive database of human E3 ubiquitin ligases: application to aquaporin-2 regulation. Physiol. Genomics 48 (7), 502–512. 10.1152/physiolgenomics.00031.2016 27199454 PMC4967219

[B98] Meng L. Person R. E. Beaudet A. L. (2012). Ube3a-ATS is an atypical RNA polymerase II transcript that represses the paternal expression of Ube3a. Hum. Mol. Genet. 21 (13), 3001–3012. 10.1093/hmg/dds130 22493002 PMC3465693

[B99] Meng L. Person R. E. Huang W. Zhu P. J. Costa-Mattioli M. Beaudet A. L. (2013). Truncation of ube3a-ATS unsilences paternal Ube3a and ameliorates behavioral defects in the angelman syndrome mouse model. PLoS Genet. 9 (12), e1004039. 10.1371/journal.pgen.1004039 24385930 PMC3873245

[B100] Meng L. Ward A. J. Chun S. Bennett C. F. Beaudet A. L. Rigo F. (2015). Towards a therapy for Angelman syndrome by targeting a long non-coding RNA. Nature 518 (7539), 409–412. 10.1038/nature13975 25470045 PMC4351819

[B101] Milazzo C. Mientjes E. J. Wallaard I. Rasmussen S. V. Erichsen K. D. Kakunuri T. (2021). Antisense oligonucleotide treatment rescues UBE3A expression and multiple phenotypes of an Angelman syndrome mouse model. JCI Insight 6 (15), e145991. 10.1172/jci.insight.145991 34369389 PMC8410092

[B102] Miura K. Kishino T. Li E. Webber H. Dikkes P. Holmes G. L. (2002). Neurobehavioral and electroencephalographic abnormalities in Ube3aMaternal-deficient mice. Neurobiol. Dis. 9 (2), 149–159. 10.1006/nbdi.2001.0463 11895368

[B103] Miura Y. Li M. Y. Revah O. Yoon S. J. Narazaki G. Pașca S. P. (2022). Engineering brain assembloids to interrogate human neural circuits. Nat. Protoc. 17 (1), 15–35. 10.1038/s41596-021-00632-z 34992269

[B104] Nazor K. L. Altun G. Lynch C. Tran H. Harness J. V. Slavin I. (2012). Recurrent variations in DNA methylation in human pluripotent stem cells and their differentiated derivatives. Cell. Stem Cell. 10 (5), 620–634. 10.1016/j.stem.2012.02.013 22560082 PMC3348513

[B105] Neureiter A. Brändl B. Hiber M. Tandon R. Müller F. J. Steenpass L. (2018). Generation of an iPSC line of a patient with Angelman syndrome due to an imprinting defect. Stem Cell. Res. 33, 20–24. 10.1016/j.scr.2018.09.015 30296670

[B106] NHS (2023). Angelman syndrome. Available at: https://www.nhs.uk/conditions/angelman-syndrome/ (Accessed: July 7, 2023).

[B107] Niki T. Imamura K. Enami T. Kinoshita M. Inoue H. (2019). Establishment of human induced pluripotent stem cell line from a patient with Angelman syndrome carrying the deletion of maternal chromosome 15q11.2-q13. Stem Cell. Res. 34, 101363. 10.1016/j.scr.2018.101363 30605843

[B108] Norat P. Soldozy S. Sokolowski J. D. Gorick C. M. Kumar J. S. Chae Y. (2020). Mitochondrial dysfunction in neurological disorders: exploring mitochondrial transplantation. npj Regen. Med. 5 (1), 22. 10.1038/s41536-020-00107-x 33298971 PMC7683736

[B109] O’Geen H. Beitnere U. Garcia M. S. Adhikari A. Cameron D. L. Fenton T. A. (2023). Transcriptional reprogramming restores UBE3A brain-wide and rescues behavioral phenotypes in an Angelman syndrome mouse model. Mol. Ther. 31 (4), 1088–1105. 10.1016/j.ymthe.2023.01.013 36641623 PMC10124086

[B110] Pandya N. J. Wang C. Costa V. Lopatta P. Meier S. Zampeta F. I. (2021). Secreted retrovirus-like GAG-domain-containing protein PEG10 is regulated by UBE3A and is involved in Angelman syndrome pathophysiology. Cell. Rep. Med. 2 (8), 100360. 10.1016/j.xcrm.2021.100360 34467244 PMC8385294

[B111] Pasca S. P. Arlotta P. Bateup H. S. Camp J. G. Cappello S. Gage F. H. (2022). A nomenclature consensus for nervous system organoids and assembloids. Nature 609 (7929), 907–910. 10.1038/s41586-022-05219-6 36171373 PMC10571504

[B112] Paşca S. P. (2019). Assembling human brain organoids. Science 363 (6423), 126–127. 10.1126/science.aau5729 30630918

[B113] Pastuzyn E. D. Shepherd J. D. (2017). Activity-dependent arc expression and homeostatic synaptic plasticity are altered in neurons from a mouse model of angelman syndrome. Front. Mol. Neurosci. 10, 234. 10.3389/fnmol.2017.00234 28804447 PMC5532393

[B114] Pham A. Selenou C. Giabicani E. Fontaine V. Marteau S. Brioude F. (2022). Maintenance of methylation profile in imprinting control regions in human induced pluripotent stem cells. Clin. Epigenetics 14 (1), 190. 10.1186/s13148-022-01410-8 36578048 PMC9798676

[B115] Pólvora-Brandão D. Joaquim M. Godinho I. Aprile D. Álvaro A. R. Onofre I. (2018). Loss of hierarchical imprinting regulation at the Prader-Willi/Angelman syndrome locus in human iPSCs. Hum. Mol. Genet. 27 (23), 3999–4011. 10.1093/hmg/ddy274 30102380 PMC6240739

[B116] Puffenberger E. G. Jinks R. N. Wang H. Xin B. Fiorentini C. Sherman E. A. (2012). A homozygous missense mutation in *HERC2* associated with global developmental delay and autism spectrum disorder. Hum. Mutat. 33 (12), 1639–1646. 10.1002/humu.22237 23065719

[B117] Qian J. Guan X. Xie B. Xu C. Niu J. Tang X. (2023). Multiplex epigenome editing of *MECP2* to rescue Rett syndrome neurons. Sci. Transl. Med. 15 (679), eadd4666. 10.1126/scitranslmed.add4666 36652535 PMC11975455

[B118] Reis A. Dittrich B. Greger V. Buiting K. Lalande M. Gillessen-Kaesbach G. (1994). Imprinting mutations suggested by abnormal DNA methylation patterns in familial Angelman and Prader-Willi syndromes. Am. J. Hum. Genet. 54 (5), 741–747. 8178815 PMC1918261

[B119] Robb S. A. Pohl K. R. Baraitser M. Wilson J. Brett E. M. (1989). The “happy puppet” syndrome of Angelman: review of the clinical features. Archives Dis. Child. 64 (1), 83–86. 10.1136/adc.64.1.83 PMC1791803 2466440

[B120] Rotaru D. C. van Woerden G. M. Wallaard I. Elgersma Y. (2018). Adult *Ube3a* gene reinstatement restores the electrophysiological deficits of prefrontal cortex layer 5 neurons in a mouse model of angelman syndrome. J. Neurosci. 38 (37), 8011–8030. 10.1523/JNEUROSCI.0083-18.2018 30082419 PMC6596147

[B121] Rotaru D. C. Wallaard I. de Vries M. van der Bie J. Elgersma Y. (2023). UBE3A expression during early postnatal brain development is required for proper dorsomedial striatal maturation. JCI Insight 8 (4), e166073. 10.1172/jci.insight.166073 36810252 PMC9977510

[B122] Rougeulle C. Cardoso C. Fontés M. Colleaux L. Lalande M. (1998). An imprinted antisense RNA overlaps UBE3A and a second maternally expressed transcript. Nat. Genet. 19 (1), 15–16. 10.1038/ng0598-15 9590281

[B123] Rougeulle C. Glatt H. Lalande M. (1997). The Angelman syndrome candidate gene, UBE3A/E6-AP, is imprinted in brain. Nat. Genet. 17 (1), 14–15. 10.1038/ng0997-14 9288088

[B124] Runte M. Hüttenhofer A. Gross S. Kiefmann M. Horsthemke B. Buiting K. (2001). The IC-SNURF-SNRPN transcript serves as a host for multiple small nucleolar RNA species and as an antisense RNA for UBE3A. Hum. Mol. Genet. 10 (23), 2687–2700. 10.1093/hmg/10.23.2687 11726556

[B125] Sadikovic B. Fernandes P. Zhang V. W. Ward P. A. Miloslavskaya I. Rhead W. (2014). Mutation update for UBE3A variants in angelman syndrome. Hum. Mutat. 35 (12), 1407–1417. 10.1002/humu.22687 25212744

[B126] Santini E. Turner K. L. Ramaraj A. B. Murphy M. P. Klann E. Kaphzan H. (2015). Mitochondrial superoxide contributes to hippocampal synaptic dysfunction and memory deficits in angelman syndrome model mice. J. Neurosci. 35 (49), 16213–16220. 10.1523/JNEUROSCI.2246-15.2015 26658871 PMC4682786

[B127] Schmid R. S. Deng X. Panikker P. Msackyi M. Breton C. Wilson J. M. (2021). CRISPR/Cas9 directed to the Ube3a antisense transcript improves Angelman syndrome phenotype in mice. J. Clin. Investigation 131 (5), e142574. 10.1172/JCI142574 PMC7919720 33411694

[B128] Sen D. Voulgaropoulos A. Drobna Z. Keung A. J. (2020). Human cerebral organoids reveal early spatiotemporal dynamics and pharmacological responses of UBE3A. Stem Cell. Rep. 15 (4), 845–854. 10.1016/j.stemcr.2020.08.006 PMC7561513 32916124

[B129] Shemer R. Hershko A. Y. Perk J. Mostoslavsky R. Tsuberi B. Cedar H. (2000). The imprinting box of the Prader-Willi/Angelman syndrome domain. Nat. Genet. 26 (4), 440–443. 10.1038/82571 11101841

[B130] Silva-Santos S. van Woerden G. M. Bruinsma C. F. Mientjes E. Jolfaei M. A. Distel B. (2015). Ube3a reinstatement identifies distinct developmental windows in a murine Angelman syndrome model. J. Clin. Investigation 125 (5), 2069–2076. 10.1172/JCI80554 PMC4463212 25866966

[B131] Simchi L. Gupta P. K. Feuermann Y. Kaphzan H. (2023). Elevated ROS levels during the early development of Angelman syndrome alter the apoptotic capacity of the developing neural precursor cells. Mol. Psychiatry. 10.1038/s41380-023-02038-7 PMC10611580 36991133

[B132] Sirois C. L. Bloom J. E. Fink J. J. Gorka D. Keller S. Germain N. D. (2020). Abundance and localization of human UBE3A protein isoforms. Hum. Mol. Genet. 29 (18), 3021–3031. 10.1093/hmg/ddaa191 32833011 PMC7645711

[B133] Stanurova J. Neureiter A. Hiber M. de Oliveira Kessler H. Stolp K. Goetzke R. (2016). Angelman syndrome-derived neurons display late onset of paternal UBE3A silencing. Sci. Rep. 6, 30792. 10.1038/srep30792 27484051 PMC4971516

[B134] Su H. Fan W. Coskun P. E. Vesa J. Gold J. A. Jiang Y. H. (2011). Mitochondrial dysfunction in CA1 hippocampal neurons of the UBE3A deficient mouse model for Angelman syndrome. Neurosci. Lett. 487 (2), 129–133. 10.1016/j.neulet.2009.06.079 19563863 PMC2888840

[B135] Sun A. X. Yuan Q. Fukuda M. Yu W. Yan H. Lim G. G. Y. (2019). Potassium channel dysfunction in human neuronal models of Angelman syndrome. Science 366 (6472), 1486–1492. 10.1126/science.aav5386 31857479 PMC7735558

[B136] Sun J. Liu Y. Moreno S. Baudry M. Bi X. (2015). Imbalanced mechanistic target of rapamycin C1 and C2 activity in the cerebellum of angelman syndrome mice impairs motor function. J. Neurosci. 35 (11), 4706–4718. 10.1523/JNEUROSCI.4276-14.2015 25788687 PMC4363395

[B137] Sun J. Liu Y. Tran J. O'Neal P. Baudry M. Bi X. (2016). mTORC1–S6K1 inhibition or mTORC2 activation improves hippocampal synaptic plasticity and learning in Angelman syndrome mice. Cell. Mol. Life Sci. 73 (22), 4303–4314. 10.1007/s00018-016-2269-z 27173058 PMC5056144

[B138] Sun J. Zhu G. Liu Y. Standley S. Ji A. Tunuguntla R. (2015). UBE3A regulates synaptic plasticity and learning and memory by controlling SK2 channel endocytosis. Cell. Rep. 12 (3), 449–461. 10.1016/j.celrep.2015.06.023 26166566 PMC4520703

[B139] Takahashi K. Tanabe K. Ohnuki M. Narita M. Ichisaka T. Tomoda K. (2007). Induction of pluripotent stem cells from adult human fibroblasts by defined factors. Cell. 131 (5), 861–872. 10.1016/j.cell.2007.11.019 18035408

[B140] Takahashi K. Yamanaka S. (2006). Induction of pluripotent stem cells from mouse embryonic and adult fibroblast cultures by defined factors. Cell. 126 (4), 663–676. 10.1016/j.cell.2006.07.024 16904174

[B141] Takahashi Y. Wu J. Suzuki K. Martinez-Redondo P. Li M. Liao H. K. (2017). Integration of CpG-free DNA induces *de novo* methylation of CpG islands in pluripotent stem cells. Science 356 (6337), 503–508. 10.1126/science.aag3260 28473583 PMC5654639

[B142] Tan W. H. Bird L. M. (2016). Angelman syndrome: current and emerging therapies in 2016. Am. J. Med. Genet. Part C Seminars Med. Genet. 172 (4), 384–401. 10.1002/ajmg.c.31536 27860204

[B143] Tang X. Jaenisch R. Sur M. (2021). The role of GABAergic signalling in neurodevelopmental disorders. Nat. Rev. Neurosci. 22 (5), 290–307. 10.1038/s41583-021-00443-x 33772226 PMC9001156

[B144] Tenreiro M. F. Branco M. A. Cotovio J. P. Cabral J. M. S. Fernandes T. G. Diogo M. M. (2023). Advancing organoid design through co-emergence, assembly, and bioengineering. Trends Biotechnol. 41 (7), 923–938. 10.1016/j.tibtech.2022.12.021 36653200

[B145] Thomson J. A. Itskovitz-Eldor J. Shapiro S. S. Waknitz M. A. Swiergiel J. J. Marshall V. S. (1998). Embryonic stem cell lines derived from human blastocysts. Science 282 (5391), 1145–1147. 10.1126/science.282.5391.1145 9804556

[B146] Valente K. D. Varela M. C. Koiffmann C. P. Andrade J. Q. Grossmann R. Kok F. (2013). Angelman syndrome caused by deletion: a genotype–phenotype correlation determined by breakpoint. Epilepsy Res. 105 (1–2), 234–239. 10.1016/j.eplepsyres.2012.12.005 23352739

[B147] Vandereyken K. Sifrim A. Thienpont B. Voet T. (2023). Methods and applications for single-cell and spatial multi-omics. Nat. Rev. Genet. 24 (8), 494–515. 10.1038/s41576-023-00580-2 36864178 PMC9979144

[B148] Vanlerberghe C. Petit F. Malan V. Vincent-Delorme C. Bouquillon S. Boute O. (2015). 15q11.2 microdeletion (BP1–BP2) and developmental delay, behaviour issues, epilepsy and congenital heart disease: a series of 52 patients. Eur. J. Med. Genet. 58 (3), 140–147. 10.1016/j.ejmg.2015.01.002 25596525

[B149] Vu T. H. Hoffman A. R. (1997). Imprinting of the Angelman syndrome gene, UBE3A, is restricted to brain. Nat. Genet. 17 (1), 12–13. 10.1038/ng0997-12 9288087

[B150] Wallace M. L. Burette A. C. Weinberg R. J. Philpot B. D. (2012). Maternal loss of Ube3a produces an excitatory/inhibitory imbalance through neuron type-specific synaptic defects. Neuron 74 (5), 793–800. 10.1016/j.neuron.2012.03.036 22681684 PMC3372864

[B151] Weston K. P. Gao X. Zhao J. Kim K. S. Maloney S. E. Gotoff J. (2021). Identification of disease-linked hyperactivating mutations in UBE3A through large-scale functional variant analysis. Nat. Commun. 12 (1), 6809. 10.1038/s41467-021-27156-0 34815418 PMC8635412

[B152] Williams C. A. Beaudet A. L. Clayton-Smith J. Knoll J. H. Kyllerman M. Laan L. A. (2006). Angelman syndrome 2005: updated consensus for diagnostic criteria. Am. J. Med. Genet. Part A 140A (5), 413–418. 10.1002/ajmg.a.31074 16470747

[B153] Williams C. A. Driscoll D. J. Dagli A. I. (2010). Clinical and genetic aspects of Angelman syndrome. Genet. Med. 12 (7), 385–395. 10.1097/GIM.0b013e3181def138 20445456

[B154] Williams C. A. Frias J. L. Opitz J. M. (1982). The angelman (“Happy puppet”) syndrome. Am. J. Med. Genet. 11 (4), 453–460. 10.1002/ajmg.1320110411 7091188

[B155] Williams C. A. Zori R. T. Stone J. W. Gray B. A. Cantu E. S. Ostrer H. (1990). Maternal origin of 15q11-13 deletions in Angelman syndrome suggests a role for genomic imprinting. Am. J. Med. Genet. 35 (3), 350–353. 10.1002/ajmg.1320350308 2309781

[B156] Wolter J. M. Mao H. Fragola G. Simon J. M. Krantz J. L. Bazick H. O. (2020). Cas9 gene therapy for Angelman syndrome traps Ube3a-ATS long non-coding RNA. Nature 587 (7833), 281–284. 10.1038/s41586-020-2835-2 33087932 PMC8020672

[B157] Wu Y. Bolduc F. V. Bell K. Tully T. Fang Y. Sehgal A. (2008). A *Drosophila* model for Angelman syndrome. Proc. Natl. Acad. Sci. 105 (34), 12399–12404. 10.1073/pnas.0805291105 18701717 PMC2527923

[B158] Xing L. Simon J. M. Ptacek T. S. Yi J. J. Loo L. Mao H. (2023). Autism-linked UBE3A gain-of-function mutation causes interneuron and behavioral phenotypes when inherited maternally or paternally in mice. Cell. Rep. 42 (7), 112706. 10.1016/j.celrep.2023.112706 37389991 PMC10530456

[B159] Yang L. Shu X. Mao S. Wang Y. Du X. Zou C. (2021). Genotype–phenotype correlations in angelman syndrome. Genes 12 (7), 987. 10.3390/genes12070987 34203304 PMC8304328

[B160] Yashiro K. Riday T. T. Condon K. H. Roberts A. C. Bernardo D. R. Prakash R. (2009). Ube3a is required for experience-dependent maturation of the neocortex. Nat. Neurosci. 12 (6), 777–783. 10.1038/nn.2327 19430469 PMC2741303

[B161] Zhang B. Korolj A. Lai B. F. L. Radisic M. (2018). Advances in organ-on-a-chip engineering. Nat. Rev. Mater. 3 (8), 257–278. 10.1038/s41578-018-0034-7

[B162] Zhou Y. Song H. Ming G. (2023). Genetics of human brain development. Nat. Rev. Genet. 10.1038/s41576-023-00626-5 PMC10926850 37507490

